# Accounting for ALA Natural Mutations Enhances the Efficiency of Graphene Oxide Nanopriming in *Bar*‐Modified *Arabidopsis*

**DOI:** 10.1002/advs.202500058

**Published:** 2025-06-05

**Authors:** Yining Wu, Rui Sun, Yueting Cui, Jun Qiao, Chengdong Zhang

**Affiliations:** ^1^ School of Environment Beijing Normal University 19 Xinjiekouwai Street, Haidian District Beijing 100875 China; ^2^ Engineering Research Center of Coal‐Based Ecological Carbon Sequestration Technology of the Ministry of Education Key Laboratory of Graphene Forestry Application of National Forest and Grass Administration Shanxi Datong University Xingyun Street, Datong City Shanxi 037009 China

**Keywords:** graphene oxide, genetically modified plants, natural variation, nanopriming, stress resilience

## Abstract

Environmental stress poses significant challenges to global agriculture, highlighting the need for resilient crops with optimized stress responses. Nanopriming and genetic modification technologies offer promising solutions. However, the differential effects of nanopriming on genetically modified (GM) and wild‐type (WT) plants remain unexplored. Using natural α‐linolenic acid (ALA) metabolism mutations as a reference, genetic variation influences resilience under graphene oxide (GO) priming is examined in *Arabidopsis thaliana*. GO priming increased chlorophyll content by 91.6% in WT plants and 29.6% in GM (*bar*‐gene) plants. Photosynthetic efficiency and quantum yield improved in the WT plants but declined in the GM plants. ALA metabolism mutations exacerbate lipid metabolism disruptions in GM plants, including altered jasmonic acid signaling and lipid composition, which compromise chloroplast integrity. Notably, transgenerational effects are observed in GM plants, with F1 seeds demonstrating dynamic epigenetic regulation of ALA metabolic genes, underscoring the influence of priming on stress resilience across generations. ALA supplementation enhanced the photosynthetic performance and chlorophyll content in WT and GM plants, with the GM plants exhibiting a significant increase of ≈60%. These findings emphasize the need for tailored nanopriming strategies that consider genetic variation and metabolic trade‐offs, thereby advancing the development of resilient crops for sustainable agriculture.

## Graphical abstract

Nanopriming enhances plant resilience to environmental challenges; however, its effectiveness across diverse genetic backgrounds, including genetic modifications and natural variations, remains uncertain. These findings demonstrate that nanopriming improves efficacy when natural genetic variations are considered, particularly in genetically modified plants. This study provides insights into the integration of nanotechnology and genetic modification for sustainable and targeted advancements.

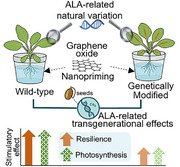

## 1 Introduction

Environmental stresses, including drought, flooding, extreme temperatures, and overuse of agricultural chemicals, significantly challenge global agriculture and affect crop growth, productivity, and food security.^[^ ^1^ ^]^ According to a report by the Food and Agriculture Organization, extreme weather events account for an average of 26% of annual global crop production losses, affecting over 34 million people worldwide.^[^ ^2^ ^]^ Building crop resilience is essential to ensure sustainable food production.^[^ ^3^ ^]^ Various technologies have been explored to enhance stress tolerance, ranging from genetic modification to innovative strategies, such as nanopriming.^[^ ^4^, ^5^, ^6^ ^]^ These methods aim to improve plant adaptability, protect yields under adverse conditions, and secure agricultural productivity in the face of increasing environmental challenges.

Nanopriming aims to expose plants to various nanomaterials, while inducing mild and controlled stress. It triggers a primed state that enhances defense responses against subsequent, more severe stresses such as drought,salinity, or pathogen attacks.^[^ ^7^ ^]^ This preparatory mechanism is distinct from harmful nanotoxicity, as it stimulates adaptive responses without causing significant physiological damage. This mechanism involves the activation of key pathways including antioxidant defense, hormone regulation, osmotic balance, and other stress‐responsive processes.^[^ ^5^ ^]^ Graphene oxide (GO) is a key nanomaterial in nanopriming that has been widely explored for enhancing crop resilience and productivity, and its agricultural use is expected to grow.^[^ ^8^ ^]^ Recently, we demonstrated that nanopriming with carbon‐based nanomaterials such as GO and nanotubes mimicked natural mechanical stimuli and enhanced pathogen resistance in *Arabidopsis thaliana* (*A. thaliana*) by ≈29%, highlighting its potential to strengthen plant immune response and resilience.^[^ ^9^ ^]^ Limited knowledge exists on how genetic diversity, including GM and natural mutations, affects the susceptibility to nanopriming.

Genetically modified (GM) plants represent another strategy for boosting stress resilience by introducing genes that enhance tolerance to environmental challenges.^[^ ^10^ ^]^ ≈206.3 million hectares of GM crops have been cultivated globally as of 2023.^[^ ^11^ ^]^ However, introducing exogenous genes can result in trade‐offs that may affect metabolism, growth, and reproduction because of resource reallocation or changes in regulatory networks.^[^ ^4^ ^]^ For instance, compared to their wild‐type (WT) counterparts, *Bt*‐modified rice has been observed to alter root exudate composition, modulate the rhizosphere microbiome, and subsequently affect soil carbon‐nitrogen metabolism.^[^ ^12^ ^]^ The *bar* gene, commonly used for herbicide resistance, can acetylate endogenous amino acids, such as aminoadipate and tryptophan, accumulating acetylated products in transgenic plants.^[^ ^13^ ^]^ Investigating whether the nanopriming patterns observed in WT plants are consistent with those in their GM counterparts is important for integrating nanopriming with genetic engineering to ensure compatibility and synergy.

Given the maturity of genetic modification technology, widely cultivated traits such as the glufosinate resistance *bar* gene generally result in stable target traits with minimal unintended physiological variations.^[^ ^14^ ^]^ In contrast, natural mutations may serve as more nuanced reference points for assessing the effects of GM on plant traits, as they typically exhibit greater diversity in non‐target traits shaped by long‐term adaptive processes.^[^ ^15^, ^16^, ^17^ ^]^ Natural mutations in α‐linolenic acid (ALA) metabolism are valuable markers due to their functional role and sensitivity to environmental stresses.^[^ ^18^ ^]^ ALA, an essential omega‐3 fatty acid and a precursor of jasmonic acid (JA), enhances biotic and abiotic stress resilience by modulating defense pathways, boosting antioxidant activity, and maintaining membrane stability under drought, salinity, and extreme temperatures.^[^ ^18^, ^19^ ^]^ Natural variations in ALA metabolism genes are also prevalent in plants and are driven by evolutionary pressures from diverse environmental conditions.^[^ ^20^ ^]^ Furthermore, genetic engineering and nanopriming inadvertently influence ALA metabolism. For example, CRISPR/Cas9 gene editing has been used to modify the *FAE1* gene in oilseed crops, indirectly increasing ALA content by altering the overall fatty acid composition.^[^ ^21^ ^]^ Treating alfalfa with graphene (5 g kg^−1^) for 35 days significantly affected ALA metabolism.^[^ ^22^ ^]^ Lastly, natural mutations in the ALA metabolism can lead to flawed stress responses and altered photosynthetic activity, representing a discernible biological phenotypic alteration derived from genotypic mutations. ALA metabolism drives chlorophyll biosynthesis, directly influencing the capacity of plants to absorb light and convert it into energy.^[^ ^18^ ^]^ Therefore, by leveraging natural mutations in ALA metabolism and widely cultivated *bar*‐modified crops, we aimed to assess the differential responses of GM and WT plants to nanopriming under natural genetic variations, providing insights relevant to real‐world agricultural scenarios.

Nanopriming significantly enhanced the photosynthetic activity and chlorophyll content in WT plants. However, it was less effective in GM plants because of amplified disruptions in ALA metabolism, which resulted in suboptimal photosynthetic efficiency and chloroplast shrinkage. ALA supplementation effectively mitigated these effects, enhancing photosynthetic activity and increasing chlorophyll content, with a particularly pronounced boosting effect of up to 60% in GM plants. GO‐induced memory, inherited through epigenetic modifications in seed methylation, highlights the profound impact of nanopriming on GM compared to WT plants across generations. This study highlights the importance of natural genetic variation in influencing plant responses to nanotechnology, particularly in GM plants. Our findings indicate that combining metabolic manipulation with nanopriming can improve crop resilience, thereby offering a focused method for enhancing agricultural performance under environmental stress.

## 2 Results and Discussion

### 2.1 Consistent and Inheritable Natural Mutations Associated with ALA Metabolism

**Figure 1:**
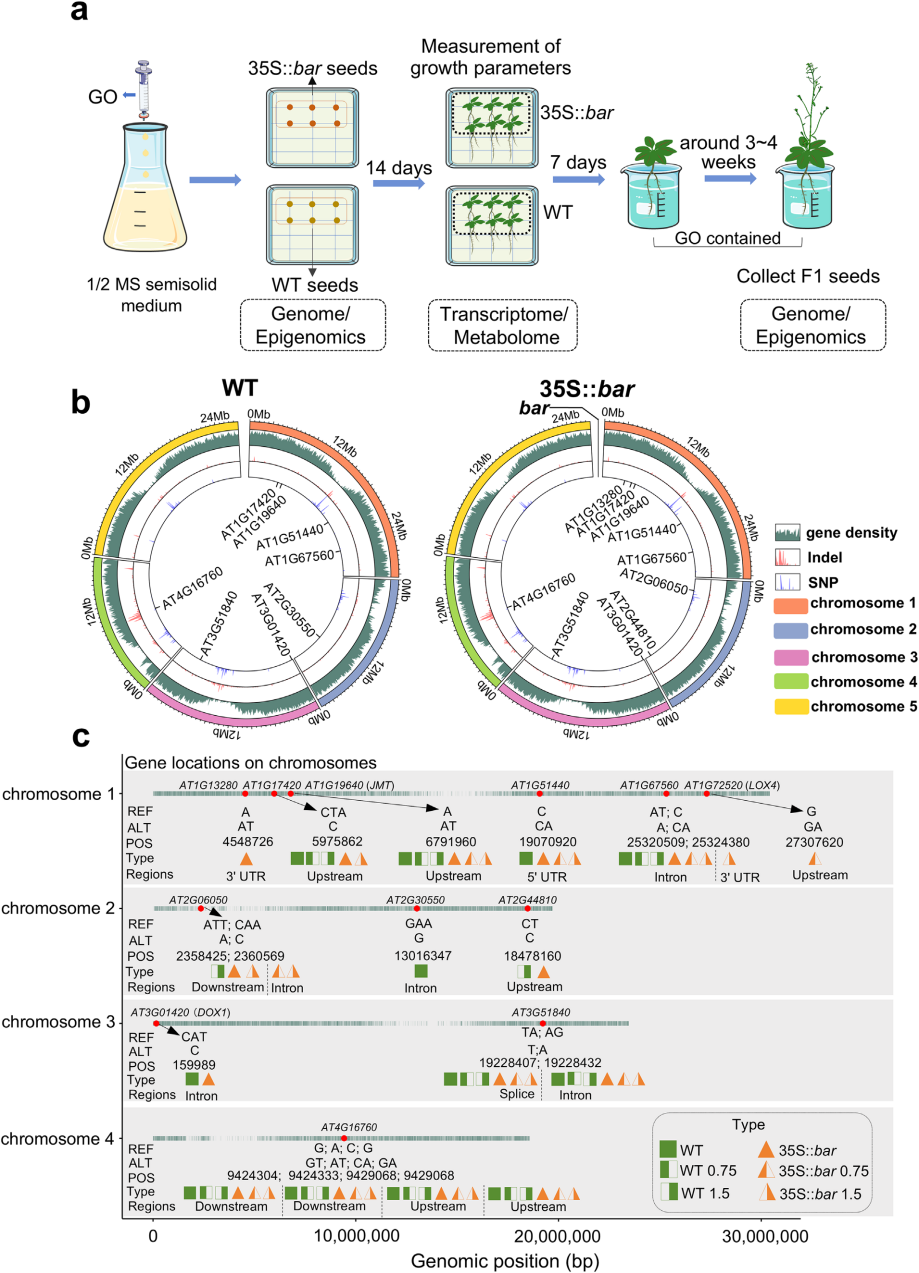
Experimental setup and genomic mutation analysis in WT and GM plants. a) Schematic of the experimental workflow. b) Circos plot showing the distribution of SNPs and Indels across chromosomes in seeds of WT and GM plants from the ancestral generation. Colored sections on the outer ring represent different chromosomes. The green inner ring indicates the density of genes across the genome. Red and blue lines represent the densities of Indels and SNPs, respectively. Density is defined as the number of SNPs/Indels per 100 kb. The central labels indicate ALA metabolism‐related genes within the genome. c) Mutation patterns in ALA metabolism‐related genes across WT, GM ancestor, and F1 seeds after GO treatment. REF and ALT indicate reference and mutated nucleotides, respectively, at specific genomic positions (POS). Type represents the categories of samples; WT and 35S::*bar* correspond to the WT and GM ancestor, respectively. F1 seeds treated with 0.75 and 1.5 mg‐C/L GO are labeled as WT 0.75, WT 1.5, 35S::*bar* 0.75, and 35S::*bar* 1.5. Regions specify functional contexts, including 3′ UTR, 5′ UTR, introns, upstream, downstream, and splice sites. Each treatment was conducted with three biological replicates.

**Figure** **1**a shows the experimental procedure in which WT and 35S::*bar* seeds were grown on 1/2 Murashige and Skoog semisolid medium with GO. The concentrations of 0.75 and 1.5 mg‐C/L, corresponding to ≈2 and 4 mg L^−1^ GO (based on the following composition analysis), respectively, were within the low‐dose range typically used in priming studies.^[^ ^23^ ^]^ These concentrations can induce observable biological responses such as stimulation, without necessarily causing lethal outcomes.^[^ ^24^ ^]^ The plants were subjected to transcriptomic analysis on day 14, and root metabolites were analyzed using ultra‐performance liquid chromatography‐tandem mass spectrometry (UPLC‐MS/MS). After an additional week of growth, the plants were transferred to a water vessel and continuously exposed to a consistent level of GO, with the water replaced every three days. Once mature, ancestral and offspring seeds were subjected to genomic and methylomics analyses. GM plants were developed using a well‐established transformation technique that has been commercially adopted to generate transgenic plants.^[^ ^25^ ^]^ Briefly, the GM plants contained the *bar* gene under the control of the 35S promoter, the *kana* gene for kanamycin resistance, and *GFP* as a reporter (Figure S1a, Supporting Information). The fluorescence of the flowers and leaves indicated successful *bar* gene expression (Figure S1b–e, Supporting Information). The *bar* gene modification is commercially available and is commonly used in crop agriculture, including maize, wheat, and rice.^[^ ^26^ ^]^ For GO characterization (Figure S2, Supporting Information), the sheet‐like structure was visualized using transmission electron microscopy (TEM) (Figure S2a, Supporting Information), and the typical X‐ray diffraction (XRD) peak for GO is ≈2θ–11°, corresponding to the (001) plane (Figure S2b, Supporting Information). The Intensity ratio of the D to G bands (I_D_/I_G_) of 0.91 in the Raman spectra indicates structural defects due to oxidation (Figure S2c, Supporting Information). X‐ray photoelectron spectroscopy (XPS) analysis suggested the presence of oxygen‐containing functional groups such as C≐O (286.80 eV), C─O (286.20 eV), and O─C≐O (288.80 eV) on the GO surface (Figure S2d, Supporting Information).

ALA metabolism‐related natural mutations were consistent between the WT and GM counterparts and were inherited in F1 seeds following GO priming. Figure 1b shows the natural variation in genomic mutations between the WT and GM *A. thaliana* plants. The circos plot illustrates the frequency distribution of DNA polymorphisms per 100 kb in the genome, showing single nucleotide polymorphisms (SNPs) and insertions/deletions (Indels) in chromosomes 1–5. Specifically, a consistent mutation pattern was observed in ALA metabolism‐related genes across samples from WT and GM ancestor seeds and F1 seeds after GO treatment (Figure 1c). Figure S3 (Supporting Information) shows the chromosome‐wise distribution of DNA polymorphisms in F1 seeds from WT and GM plants after GO treatment using circos plots. Figure 1c shows that *AT1G19640* (*JMT*) has an upstream variant that affects transcription, *AT1G67560* (*LOX6*) has intron variants that disrupt splicing, *AT3G51840* (*ACX4*) has splice‐site mutations, and *AT4G16760* (*ACX1*) has multiple mutations that may affect gene expression and stability; each gene exhibited a similar mutation pattern across WT and GM ancestral seeds and F1 seeds following GO treatment. Therefore, these natural mutations were inherited across generations regardless of whether the plants were treated with GO. For GM and WT seeds, scatter plots of enriched pathways (based on genomic mutations) with a gene number >5 (i.e., more than five genes involved out of the total genes in that pathway) showed that ALA metabolism and its potential impact on lipid metabolism were among the enriched pathways (Figure S4, Supporting Information).

### 2.2 GO Exacerbates ALA Metabolism Downregulation in GM vs WT Plants

**Figure 2:**
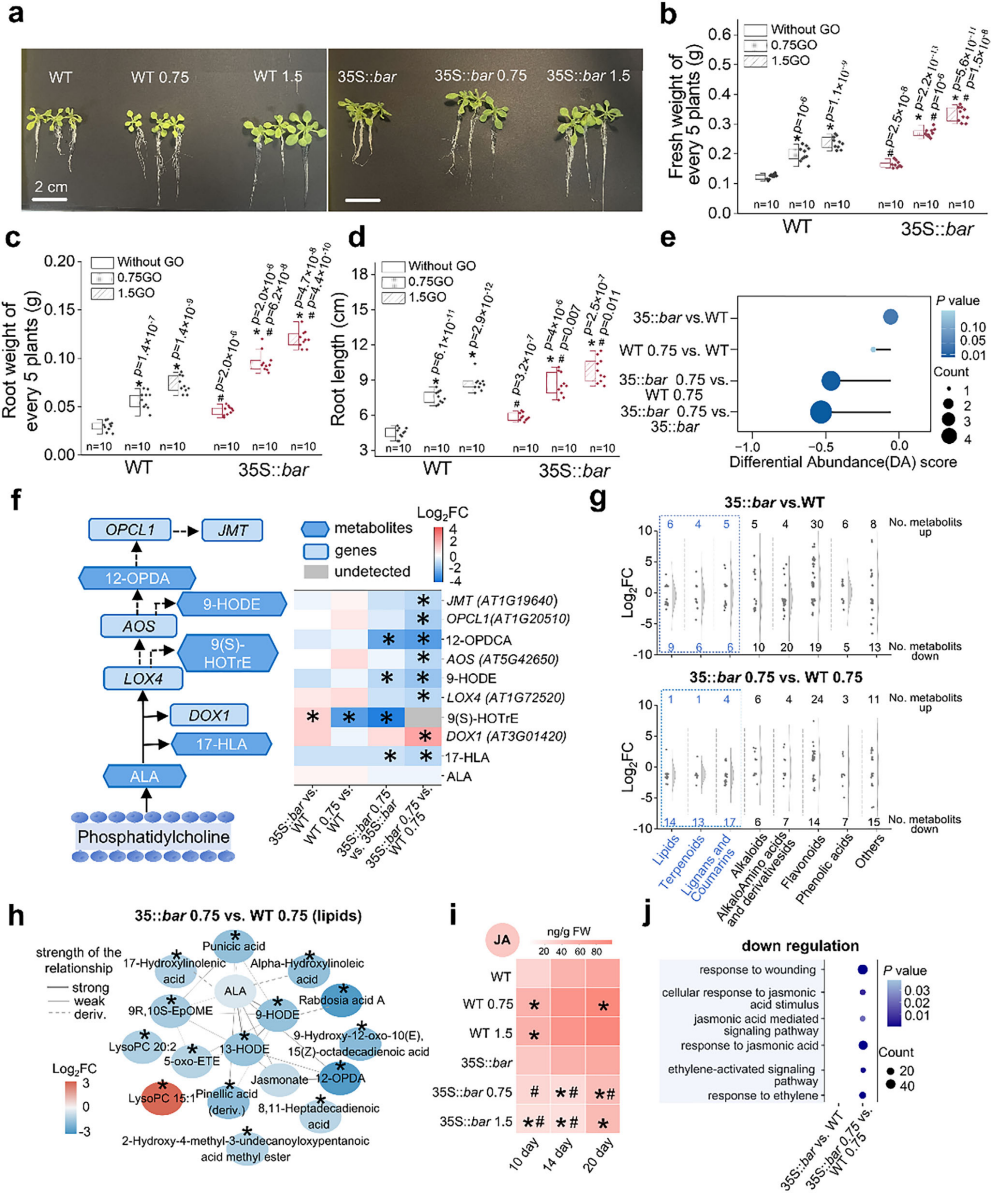
GO exacerbated ALA mutation‐related changes in GM vs WT plants. a) Representative images of 14‐day‐old WT and GM plants captured with a smartphone. Measurements of b) fresh weight, c) root weight, and d) root length. *p* values and sample size (*n* = 10) are provided in the figure. Plants were treated for 14 days with 0, 0.75, or 1.5 mg‐C/L GO. e) Differential abundance (DA) analysis of metabolomic profiles related to ALA metabolism in WT and GM plants. The x‐axis shows shifts in metabolite abundance; dot size represents the number of significant metabolites, and color intensity indicates statistical significance. Line length aids visual comparison relative to zero. f) Co‐analysis of metabolites and gene expression associated with ALA metabolism. The left panel illustrates the ALA metabolic pathway, whereas the right panel contains a heatmap of Log_2_FC values for metabolites and genes. Gene names and identifiers clarify their relationships to the mutations depicted in Figure 1b,c. g) Comparative analysis of metabolite shifts in GM and WT plants with or without GO treatment. Metabolites are categorized, with upregulated and downregulated counts displayed above and below each group, respectively. Blue markings highlight significantly downregulated metabolites. h) Lipid metabolite network analysis under 0.75 mg‐C/L GO treatment. Nodes represent lipid metabolites, color‐coded by Log_2_FC between GM and WT plants. Line intensity reflects the strength of relationships, and dotted lines denote structural derivatives. Metabolite relationship strength was inferred from STITCH interaction scores. Abbreviations for 12‐OPDA, 13‐HODE, 5‐oxo‐ETE, 9R,10S‐EpOME, 9‐HODE, LysoPC 15:1, and LysoPC 20:2 are provided in Table S1 (Supporting Information). In f), genes marked with ^*^ indicate |Log_2_FC| > 1 and a Benjamini–Hochberg adjusted *p* < 0.05. In (f,h), metabolites marked with ^*^ satisfy |Log_2_FC| > 1 and variable importance in projection (VIP) > 1. i) JA levels in the roots of WT and GM plants subjected to 0.75 and 1.5 mg‐C/L GO treatments (sample size *n* = 3). *p* values for all comparisons are provided in Table S2 (Supporting Information). j) Gene ontology enrichment analysis of co‐expressed genes and metabolites (|correlation coefficient| > 0.8, *p* < 0.05, Log_2_FC < ‐1). Pathways with Bonferroni‐corrected *p* < 0.05 are displayed; blank cells indicate no significant enrichment. Three biological replicates per group were used for transcriptome, metabolome, and JA measurements. For (a–c,i), independent sample two‐sided *t*‐tests were performed: ^*^ indicates a significant difference when comparing with WT or GM at the same time point; ^#^ indicates a significant difference compared to WT under the same GO treatment at the same time point.

After GO priming, the WT and GM plants consistently showed enhanced growth (**Figure** **2**a–d), characterized by increased biomass accumulation and improved root development. Figure 2a presents representative images of plants, while Figure 2b–d demonstrates that nanopriming significantly increases fresh weight by up to 92% in WT and 103% in GM plants, root weight by 142% in WT and 160% in GM plants, and root length by 94% in WT and 67% in GM plants. Additional physiological indicators were assessed, including the antioxidant activities, soluble sugar content, proline accumulation, and hormone levels (Figure S5, Supporting Information). Overall, nanopriming displayed varying degrees of stimulatory effects on both genotypes. However, the differences between GM and WT plants were minor and fluctuating, likely reflecting the minimized non‐target physiological variability associated with genetic modification technology.

GO priming had a significantly different impact on ALA metabolism in WT and GM plants, leading to a more pronounced downregulation in GM plants than in WT plants. Metabolomic analysis revealed that the decrease in the metabolites involved in ALA metabolism was more pronounced in the GM plants than in the WT plants after GO treatment (Figure 2e). Figure S6 (Supporting Information) shows the Kyoto Encyclopedia of Genes and Genomes (KEGG) enrichment analysis of pathways based on all significantly different metabolites across various comparison groups. In contrast, the difference between GM and WT plants without GO treatment was insignificant. This result was further supported by combined transcriptomic and metabolomic analyses (Figure 2f). Notably, although ALA availability was not compromised, the downregulation of its metabolic pathway may be attributed to natural mutations in downstream genes, such as *DOX1* (Alpha‐dioxygenase 1, *AT3G01420*), *LOX4* (Lipoxygenase 4, *AT1G72520*), *AOS* (Allene oxide synthase, *AT5G42650*), and *JMT*.

Because ALA plays a crucial role in lipid signaling and synthesis pathways, its downregulation could reduce the production of essential signaling molecules, such as stress‐responsive compounds or fatty acids derived from ALA.^[^ ^27^ ^]^ To verify this, 154 significantly different metabolites were identified; after GO exposure, the lipid, terpenoid, lignan, and coumarin levels were markedly lower in the GM group than in the WT group, whereas these differences were less pronounced without GO treatment (Figure 2g). Figure 2h shows that, through metabolite‐metabolite interaction analysis (via the STITCH database), lipid metabolism is considerably affected because ALA is a critical polyunsaturated fatty acid involved in lipid metabolism. Of the 15 identified lipids, 14 were significantly reduced in the GM compared to the WT under the same GO treatment. Among the decreased lipid metabolites, 12‐OPDA (12‐Oxo‐Phytodienoic Acid), a direct derivative of ALA produced via the oxylipin biosynthesis pathway,^[^ ^28^ ^]^ was significantly downregulated (Log_2_ Fold Change [FC] = −2.76). This metabolite is a precursor of JA, a crucial signaling molecule involved in plant stress responses.^[^ ^28^ ^]^ Additionally, 9‐HODE (9‐Hydroxy‐octadecadienoic acid, Log_2_FC = −1.60) and 13‐HODE (13‐Hydroxy‐octadecadienoic acid, Log_2_FC = −1.54) are hydroxy fatty acids produced from ALA oxidation.^[^ ^29^ ^]^ Increased LysoPC (Lysophosphatidylcholine) is mainly involved in membrane dynamics, and its involvement in chloroplasts is linked to lipid transport and signaling.^[^ ^30^ ^]^ These metabolites act as signaling molecules in plant stress responses, particularly in the defense against pathogens and oxidative damage.^[^ ^28^, ^29^, ^30^ ^]^ Upon GO exposure, terpenoids, lignans, and coumarins were significantly downregulated in GM plants compared to WT plants, suggesting less effective priming of stress responses and defense mechanisms in GM plants (Figure S7, Supporting Information).

ALA is the precursor of JA, an essential plant hormone for defense and stress responses,^[^ ^31^ ^]^ and the downregulation of ALA metabolism leads to reduced levels of JA. We monitored the dynamic changes in JA content during plant growth (Figure 2i) using liquid chromatography‐mass spectrometry (HPLC‐MS).^[^ ^9^ ^]^ Plant hormones often exist at low concentrations, requiring specific extraction methods and sensitive analytical instruments for accurate detection; therefore, they have not been precisely quantified in the aforementioned widely targeted metabolome analysis. JA levels steadily increased in the WT plants from days 10 to 20, and GO stimulated JA production. The JA levels between the WT and GM plants without GO exposure showed no noticeable difference. However, after GO exposure, JA levels in the GM plants decreased significantly and remained consistently lower than those in the WT plants under similar treatments. Transcriptomic and metabolomic co‐analyses confirmed that the JA‐ethylene pathways were more significantly downregulated in GM than in WT plants in response to GO (Figure 2j).

Considering that numerous natural mutations co‐occurred in GM and WT seeds (Figure S4, Supporting Information) and that ALA‐related variation was not the most prominent among them, the effect of GO on ALA‐related natural mutations was intriguing. Our previous studies suggested that GO may enter *A. thaliana* and trigger mechanical stress responses such as wound healing and defense mechanisms.^[^ ^9^ ^]^ This has led to a significant increase in demand for JA synthesis. Consequently, natural variations in ALA metabolism‐related genes that result in a deficiency of ALA (a key precursor of JA) would have a more pronounced effect under these conditions. However, the direct effect of GO on ALA metabolism remains unclear. Depending on the dose, GO and other nanoparticles inhibit or stimulate ALA production to various levels. For instance, modified graphene was thoroughly mixed with matrix soil at 1% and 2% (w/w) concentrations and applied to *Medicago sativa* L. seedlings. Through oxidative stress, GO exposure disrupted key metabolic pathways in *M. sativa* L., including ALA biosynthesis.^[^ ^32^ ^]^ In another study, graphene nanoparticles (5 g/kg) enhanced the biomass and chlorophyll content of *M. sativa* L. under salt stress, notably influencing fatty acid biosynthesis, including the ALA metabolic pathway.^[^ ^22^ ^]^ However, these mechanisms do not directly target ALA, and their effects are primarily indirect or secondary. Future research should explore ALA as a potentially universal marker for monitoring priming effects, focusing on its role across plant systems, stress conditions, and metabolic pathways to optimize nanopriming strategies and enhance crop resilience.

### 2.3 ALA Supplementation can Improve ALA Metabolism‐Related Chloroplast Changes and Photosynthetic Decline in GM Plants

**Figure 3:**
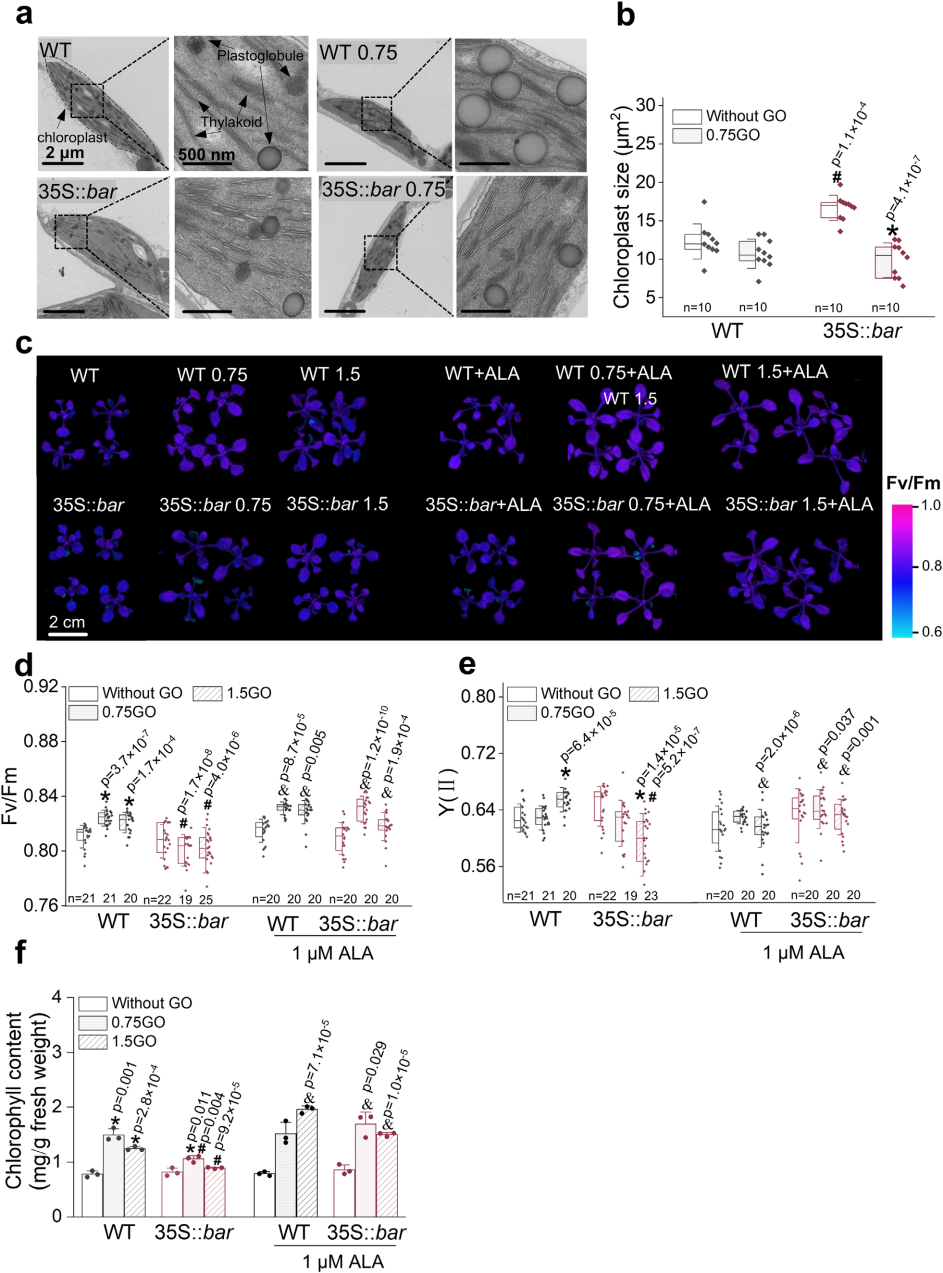
Disruption of ALA metabolism leads to impaired chloroplastic structure and reduced photosynthetic efficiency in GM plants. a) Ultrastructural comparison of chloroplasts in WT and GM plants with/without exposure to 0.75 mg‐C/L GO. Chloroplasts are highlighted with a dotted outline to visualize their shape and size. The area within the dotted outline is enlarged to show detailed chloroplast structures, with arrows pointing to thylakoid organization and plastoglobule distribution. b) Comparison of chloroplast size between WT and GM plants under 0.75 mg‐C/L GO treatment. Comparisons of Fv/Fm via chlorophyll fluorescence images c), quantification of Fv/Fm d), Y(II) e), and chlorophyll content f) in response to GO (0.75 or 1.5 mg‐C/L) with or without 1 µm ALA addition. For (b,d,e), the horizontal line within each box represents the median, the whiskers indicate the standard deviation (SD), and the box boundaries correspond to the 25th and 75th percentiles. Data in (f) are presented as means ± SD. For (b,d,e,f), gray and dark red dots represent individual data points in WT and GM groups, respectively. For (b,d,e), the sample size (n) is indicated within the figures, whereas for (f), *n* = 3, with each treatment comprising three biological replicates. Independent sample two‐sided *t*‐tests were performed: ^*^ indicates significant differences within the same genotype relative to the condition without GO; ^#^ indicates significant differences between GM and WT under the same treatment; and ^&^ indicates significant differences between the same treatment with and without ALA addition. Significant differences are marked with *p* values.

We examined the morphology of chloroplasts and their photosynthetic activity to connect genotypic variation with phenotypic outcomes, thereby providing insights into the role of ALA metabolism in stress resilience. ALA is synthesized in the chloroplasts and plays a role in maintaining membrane fluidity and stability, which are essential for optimal photosynthetic activity and chloroplast function.^[^ ^18^ ^]^ We examined chloroplast morphology using TEM, revealing slight differences between the GM and WT plants. GM chloroplasts were slightly larger than those of the WT (**Figure** **3**a), with similarly packed thylakoids and plastoglobules. Insufficient ALA metabolism compromises membrane stability and results in chloroplast shrinkage under stress.^[^ ^29^ ^]^ Collaboratively, chloroplast shrinkage was significant in GM plants upon GO exposure, with a size reduction of ≈40% (Figure 3a,b). In contrast, no pronounced shrinkage was observed in the WT chloroplasts, likely because of the significantly enlarged plastoglobules, which help maintain integrity by storing and recycling lipids, supporting membrane repair, and stabilizing thylakoid structures to preserve photosynthetic function.^[^ ^33^ ^]^ Such an enlargement of plastoglobules was not observed in the GM plants, even under GO stress, possibly because plastoglobule size is often strictly regulated by the availability of lipid precursors (e.g., ALA and related metabolites in our study).^[^ ^34^ ^]^ Therefore, in the context of ALA metabolism, GO priming was less effective in GM plants than in WT plants because natural mutations led to more severe disruptions in the pathway, specifically in GM plants.

Chloroplast shrinkage and plastoglobule dysfunction can compromise the overall function of chloroplasts, ultimately reducing their photosynthetic activity.^[^ ^35^ ^]^ Using chlorophyll fluorescence imaging, we visualized the photosynthetic efficiency of 14‐day‐old plants (Figure 3c). Analysis of the maximum quantum efficiency of Photosystem II (PSII) (Fv/Fm) under dark conditions revealed that GO treatment increased the Fv/Fm values in WT plants and slightly decreased them in GM plants (Figure 3d). No significant difference was observed between the GM and WT plants without GO priming. Supplementation with 1 µmol L^−1^ ALA mitigated the effects of GO and improved Fv/Fm in WT and GM groups. The mitigation effect of ALA was more apparent in the GM group than in the WT group for PSII quantum yield (Y(II)) (Figure 3e), which represents the efficiency of PSII in converting light into chemical energy. ALA supplementation increased the chlorophyll content in GM and WT plants (Figure 3f) by up to two‐fold, highlighting the importance of adequate ALA levels in maintaining healthy chloroplasts and stable chlorophyll levels^[^ ^36^ ^]^ particularly under GO exposure.

**Figure 4:**
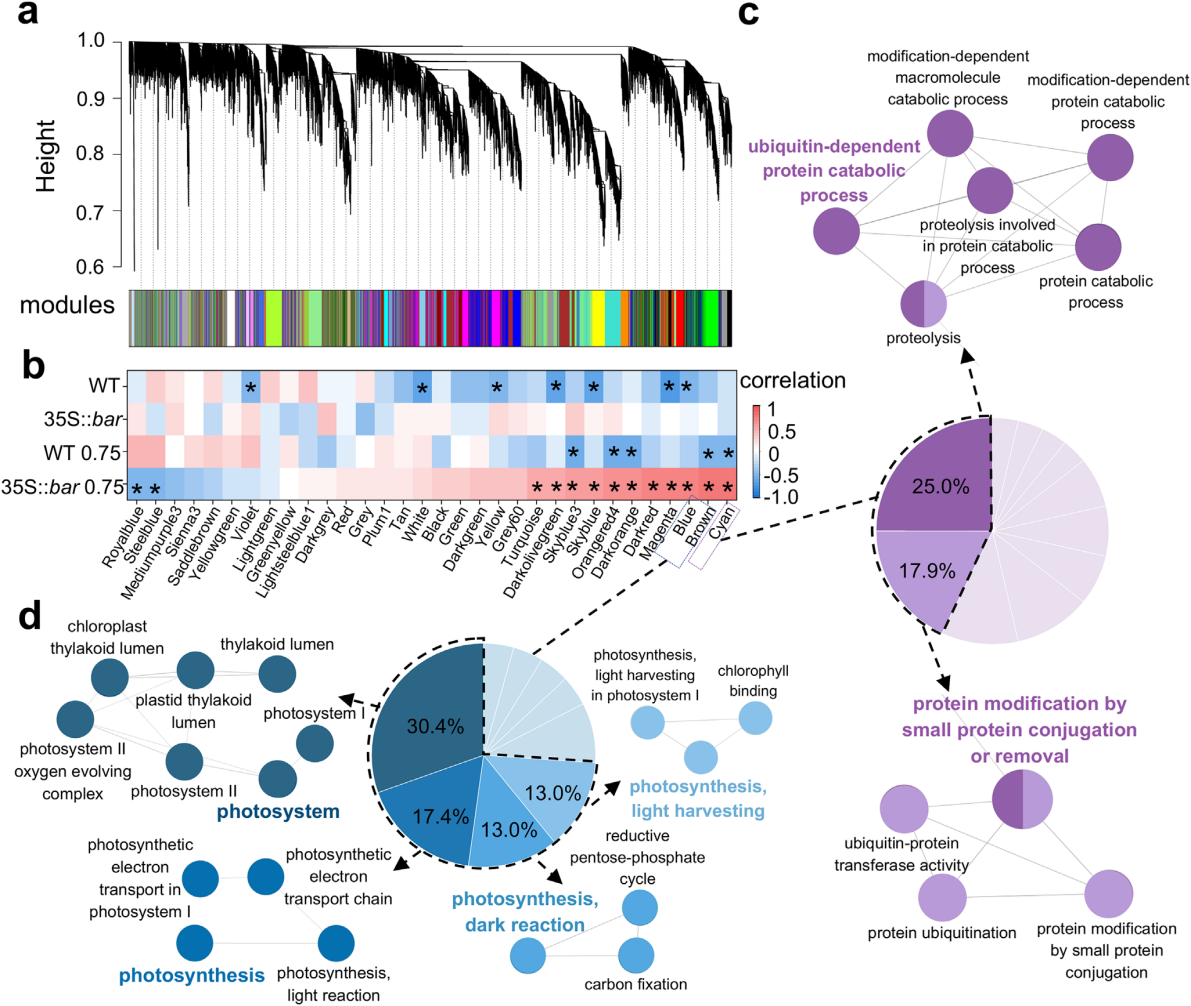
WGCNA identifies gene modules and enriched biological processes in WT and GM plants under 0.75 mg‐C/L GO exposure. a) Dendrogram of hierarchical clustering showing different gene modules represented by distinct colors. Each color represents a group of co‐expressed genes with similar expression patterns across different treatments (sample size *n* = 3). b) Heatmap displaying the correlation of different gene modules with treatments and genotypes. Positive and negative correlations are depicted in red and blue, respectively, with asterisks indicating significant correlations (*p* < 0.05). Gene modules were ranked from the most substantial negative to the most potent positive correlations in the GO‐treated GM group. Enrichment analysis of the cyan c) and brown and blue modules d). In (c,d), pie charts illustrate the proportion of enriched gene ontology clusters within each module, with different colors representing distinct gene ontology clusters and the numbers indicating the proportion of each cluster relative to the total enriched gene ontology terms. The colors of each cluster in the pie charts correspond to the colors of the GO clusters shown in the bubble plot. Only gene ontology terms with Bonferroni‐corrected term *p* < 0.05 and gene ontology clusters with Bonferroni‐corrected group *p* < 0.05 are displayed.

Impaired photosynthetic activity and stress response in GM plants following GO exposure were further characterized using weighted gene expression network analysis (WGCNA) of the transcriptomic data. **Figure** **4**a shows gene clustering based on coexpression patterns, with colored blocks representing distinct modules of genes with similar profiles. The heatmap in Figure 4b shows the correlation between the gene modules (x‐axis) and different treatments (y‐axis). Dark red indicates a high positive correlation, with the cyan (correlation coefficient = 0.79, *p* = 1 × 10^−4^), brown (correlation coefficient = 0.79, *p* = 1 ×10^−4^), and blue (correlation coefficient = 0.72, *p* = 7 × 10^−4^) modules showing the strongest positive correlations with the phenotypic features of GM plants under GO stress. Gene ontology analysis revealed that 43% of the pathways enriched in the cyan module were associated with “protein modification by small protein conjugation or removal” and “ubiquitin‐protein transferase activity,” as indicated by the purple network (Figure 4c). Protein ubiquitination and degradation are crucial for the removal of damaged proteins, particularly PSII, from photosystems under stressful conditions.^[^ ^37^ ^]^ These two biological pathways align with the roles of plastoglobules in the stress response, membrane remodeling, and lipid metabolism.^[^ ^38^ ^]^ Furthermore, >73% of the enriched pathways in the brown and blue modules were related to photosynthetic processes, particularly those involving PSII (Figure 4d). Correspondingly, under 0.75 mg‐C/L GO exposure, GM plants showed significant downregulation of thylakoid, plastid, chloroplast structure, and stress response pathways compared to WT plants, ranking among the top 25 downregulated differentially enriched pathways (Figure S8, Supporting Information). Structural and functional differences in the chloroplasts, plastoglobule size, and photosynthetic function were confirmed through transcriptome analysis.

Consistent structural alterations and transcriptomic analyses suggested that *bar*‐GM plants are more susceptible to nanopriming, particularly in response to natural ALA mutations. The *bar* gene function is confined to herbicide resistance and operates independently of the enzymatic processes governing ALA metabolism. However, the high antioxidative cost in GM plants (Figure S5, Supporting Information) may be intertwined with ALA‐related anti‐reactive oxygen species (ROS) functions, leading to metabolic tradeoffs that disrupt lipid homeostasis.^[^ ^39^ ^]^ Plants containing the *bar* gene exhibit increased antioxidative activity, which may be due to the indirect involvement of antioxidant enzymes. For instance, co‐expression of the *bar* gene with other stress‐responsive genes such as *AtNHX1* has been associated with enhanced tolerance to oxidative stress in transgenic mung bean plants.^[^ ^40^ ^]^ Moreover, the *bar* gene exhibited nonspecific activity in plants by acetylating endogenous amino acids and accumulating altered metabolic products across various species.^[^ ^13^ ^]^ However, how these unintended modifications indirectly affect metabolic pathways, including those related to antioxidant function, remains largely unexplored. Conversely, ALA‐related antioxidant functions were evident. Transcriptomic analysis of ALA‐treated *A. thaliana* revealed its role in modulating gene expression related to oxidative stress signaling and significantly enhancing ROS stress responses.^[^ ^41^ ^]^ Together, the overlapping effects of mild stress from GO priming, ALA deficiency due to natural mutations, and high oxidative cost in GM plants synergistically amplify metabolic disruptions, leading to intensified physiological stress and altered adaptive responses. Future studies should underscore the need for tailored nanopriming strategies that complement genetic variation to enhance plant resilience while minimizing metabolic tradeoffs, ultimately improving crop performance and agricultural sustainability.

### 2.4 Methylation Dynamics in ALA Metabolism Suggested a Greater Inheritable Stress Response in GM Plants than in WT Plants

**Figure 5:**
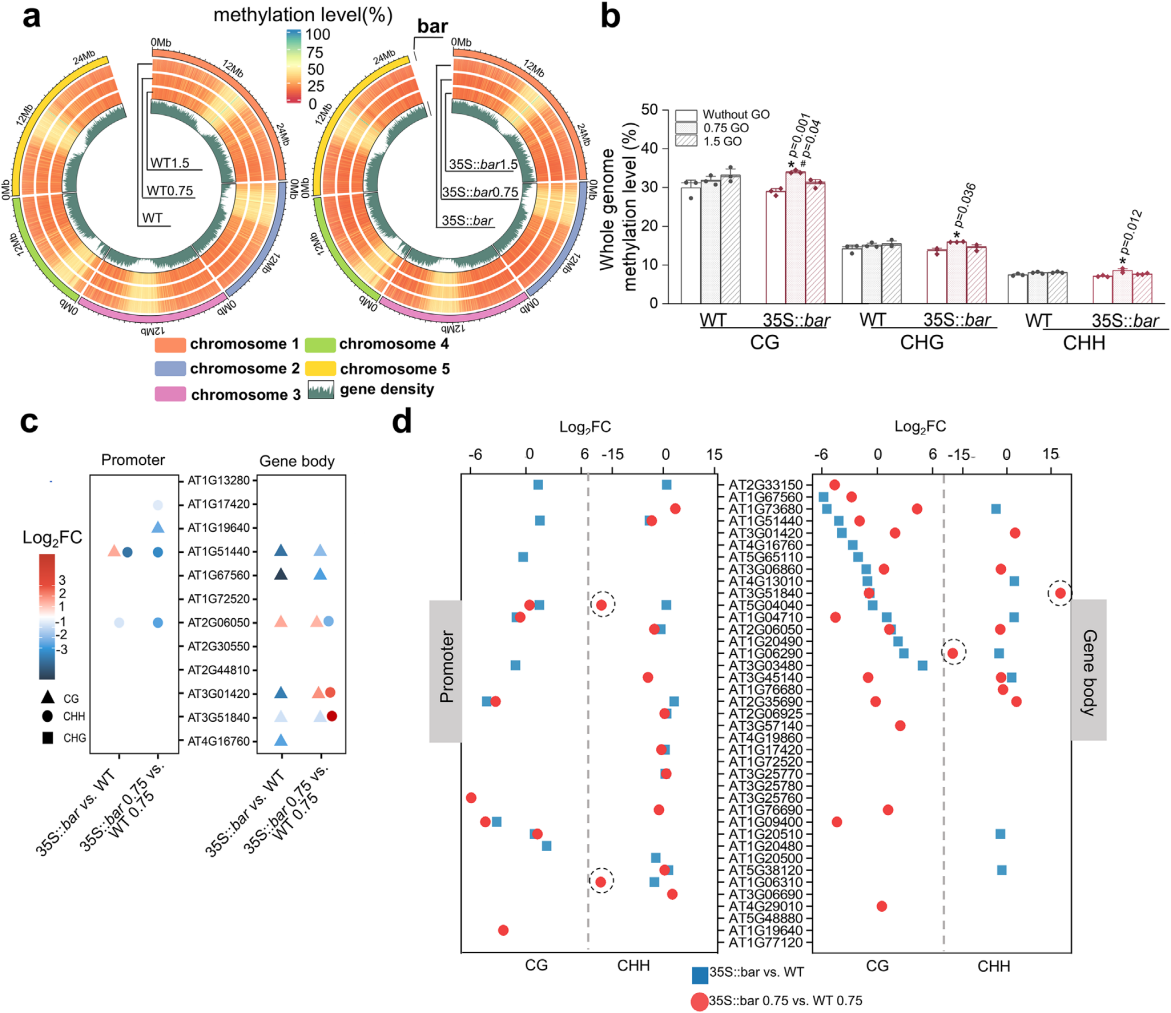
Genome‐wide DNA methylation analysis in WT and GM seeds before and after GO treatments. a) Circos plots show the methylation levels across different chromosomes for WT and GM seeds before and after GO exposure. The colored sections on the outer ring represent different chromosomes. The concentric rings from outer to inner illustrate untreated samples, 0.75 mg‐C/L GO‐treated samples, and 1.5 mg‐C/L GO‐treated samples. Whole‐genome methylation levels were calculated as mean values within 100 kb windows for each chromosome. The color gradient from cyan to red indicates the methylation percentages, whereas the innermost ring represents gene density. b) Whole‐genome methylation levels in CG, CHG, and CHH contexts for WT and GM seeds after various GO exposures (sample size *n* = 3). Data are presented as means ± SD, with gray and red dots depicting individual data points in the WT and GM groups. Three biological replicates were included for each treatment. Independent sample two‐sided *t*‐tests were performed: ^*^ indicates significant differences within the same genotype relative to the condition without GO, and ^#^ indicates significant differences between GM and WT under the same treatment. Significant differences are marked with *p* values. c) Differential methylation regions at promoter regions and gene bodies of key genes (Figure 1c) are represented by a color gradient showing Log_2_FC in methylation levels. Triangles and circles indicate CG and CHH methylation, respectively. No differential methylation was observed in the CHG context. Regions without differential methylation are blank. d) Dot plots of differential methylation for ALA metabolism‐related genes compare promoter and gene body methylation across conditions. Blue and red dots denote different comparisons, as indicated in the legend. Dots with circles indicate methylation levels with |Log_2_FC| >10.

We evaluated the methylome in ancestral GM and WT seeds (without GO exposure) and in F1 seeds collected after GO exposure to determine whether the ALA‐related stress response was more pronounced in GM seeds than in WT seeds, allowing the offspring to inherit the stress response. **Figure** **5**a shows circos plots illustrating a comparative analysis of chromosome‐wide overall methylation levels between GM and WT plants across various GO treatments. Quantification of methylation in the CG, CHG, and CHH contexts revealed minimal difference in the overall methylation levels between the WT and GM plants, even after GO exposure (Figure 5b).

Among the differentially methylated genes of key interest (Figure 1c), methylation levels were primarily decreased in the promoter regions (in the CHH context) and gene bodies (in the CG context) of GM seeds compared to the WT (Figure 5c). The reduction in methylation in the CHH context of the promoter indicates that these genes may be more actively transcribed.^[^ ^27^ ^]^ For example, *AT2G06050*, *AT1G51440*, *AT1G19640*, and *AT1G17420* encode OPDA reductase 3, a phosphatidylcholine‐hydrolyzing lipase, *JMT*, and lipoxygenase 3, respectively, all of which are crucial for JA synthesis. Therefore, reduced methylation levels in the promoter regions of these genes following GO exposure imply an active effort to compensate for ALA deficiency and enhance plant adaptation. Furthermore, variations in methylation levels (increases and decreases) in the CG and CHH contexts of the gene body were observed. These epigenetic changes suggest that GM plants exposed to GO may experience higher stress levels and adopt diverse epigenetic regulatory mechanisms to cope with the ALA metabolism deficiency resulting from natural mutations.^[^ ^42^ ^]^

We investigated whether this epigenetic regulation extended to genes involved in ALA metabolic pathways. Figure 5d shows the dynamic methylation patterns and oscillation levels of ALA metabolism‐related genes in GM and WT plants and in GO‐treated offspring. The highly dynamic CHH methylation observed in the GM plant promoter and gene body regions compared to the WT under GO exposure suggests a high level of selective stress and strong epigenetic regulation.^[^ ^43^ ^]^ For instance, *AT5G04040* (*SDP1*) encodes a triacylglycerol lipase involved in the breakdown of storage lipids,^[^ ^44^ ^]^ whereas *AT1G06310* encodes a putative acyl‐CoA oxidase.^[^ ^45^ ^]^ Low methylation in the CHH region of the promoters of these genes (Log_2_FC < −10) indicated the potential upregulation of their expression, which may enhance lipid catabolism and support fatty acid metabolism as a compensatory mechanism.^[^ ^44^ ^]^ *AT3G51840* and *AT1G06290* are also involved in fatty acid metabolism,^[^ ^46^, ^47^ ^]^ and dynamic methylation levels in the CHH region of their gene bodies (Log_2_FC > 10 or < −10) may modulate transcriptional activity, potentially affecting overall energy metabolism.^[^ ^48^ ^]^ Moreover, dynamic epigenetic regulation within the gene body region in the CG context was more pronounced in GM F1 seeds than in WT F1 seeds after GO exposure. Owing to their low frequency, the detected CHG methylation changes are not shown in the plot. Together, these strong oscillations in GM plants may reflect high selective pressure and the need for versatile fine‐tuning of gene expression as a feedback mechanism in response to GO stress.

Epigenetic regulation is crucial in WT and GM plants; however, genetic modification can introduce unique epigenetic variations, leading to differences in gene expression and potential concerns. For instance, transgenic *A. thaliana* plants expressing the *HKT1* gene exhibited altered expression patterns compared to WT plants, leading to changes in salt sensitivity.^[^ ^49^ ^]^ In WT plants, *HKT1* is predominantly expressed in the roots. In contrast, hypomethylation of the *HKT1* promoter regions in transgenic plants leads to increased *HKT1* expression in the leaves, resulting in salt‐hypersensitive phenotypes owing to the rapid accumulation of sodium ions in the leaves. Several studies have also suggested that nanomaterials have the potential to modulate plant epigenomes, thereby affecting various physiological and developmental processes. For example, exposures to zinc, copper, and γ‐iron oxide nanoparticles caused DNA methylation changes in wheat, leading to increased polymorphism rate and cytosine methylation.^[^ ^50^ ^]^ Nevertheless, few studies have explored the impact of nanomaterials on differential epigenetic patterns between GM and WT plants. Our findings revealed that nanomaterial exposure could amplify the genotypic differences between GM and WT plants at the epigenomic level. This amplification may involve crosstalk between epigenetic regulation and hormonal signaling pathways, such as JA.^[^ ^51^ ^]^ Methyl jasmonate (MeJA), a JA derivative, influences DNA methylation patterns in plants, leading to changes in the expression of genes that enhance stress responses. For instance, in *Thymus kotschyanus*, MeJA treatment confers arsenic tolerance by inducing DNA hypomethylation, which upregulates genes involved in terpenoid metabolism and activates cytochrome P450 monooxygenases.^[^ ^52^ ^]^ Similarly, in rice (*Oryza sativa*), MeJA‐induced priming leads to increased expression of defense‐related genes upon wounding, accompanied by histone modifications in the promoter regions of these genes and changes in genome‐wide DNA methylation levels.^[^ ^53^ ^]^ However, further studies are needed to elucidate the precise mechanisms by which nanomaterial–hormone interactions influence epigenetic regulation. Understanding these interactions could provide valuable insights and inform strategies for maximizing beneficial outcomes while minimizing unintended effects.

## 3 Conclusion

Overall, GO nanopriming widened the gap in adaptive efficiency between the GM and WT plants. GO exposure amplified the effects of natural genetic variations in ALA metabolism, leading to greater downregulation of ALA metabolism, lipid metabolism, downstream JA‐ethylene signaling, and an overall reduction in chlorophyll content and photosynthetic activity in GM plants than in WT plants. This underscores the need for precise nanopriming strategies that consider genetic background to optimize stress resilience, maintain metabolic stability, and support sustainable agricultural practices under environmental stress conditions.

## 4 Experimental Section

### Materials and Characterization

The GO powder was obtained from XFNANO (Nanjing, China). The morphology, crystal structure, structural features, and surface functionalization were characterized using TEM (JEM‐F200, JEOL, Tokyo, Japan), XRD (Ultima IV, Rigaku, Tokyo, Japan), Raman spectroscopy (LabRAM HR Evolution, Horiba, Kyoto, Japan), and XPS (K‐Alpha, Thermo Scientific, MA, USA). The detailed experimental procedures are provided in the Supporting Information.

### Construction of GM *A. thaliana*

Overexpression vectors for the *bar* and *GFP* genes were constructed. *Agrobacterium tumefaciens*‐mediated transformation via the floral dip method introduced these genes into Columbia WT *A. thaliana*. Detailed construction methods are provided in the Supporting Information. The successful incorporation of the *bar* transgene was confirmed by genomic DNA extraction using the DNAsecure Plant Kit (TIANGEN) following the manufacturer's instructions and verified through Sanger sequencing on an ABI3730 platform (Thermo Fisher).

### Cultivation and GO Exposure

GO powder was dispersed in water with sonication and filtered through a 0.22 µm syringe filter. The carbon content was determined via a Total Organic Carbon analyzer (TOC‐L CPH, Shimadzu). Detailed testing conditions are provided in the Supporting Information. After dilution, 0.75 or 1.5 mg‐C/L of GO were incorporated into a 1/2 Murashige and Skoog (Solarbio) semi‐solid medium (SM) containing 0.7% agar and 1% sucrose. Surface‐sterilized WT and GM seeds were aseptically sown onto the SM plate with/without GO addition. The plates were sealed and stored at 4 °C in the dark for 2 days to induce vernalization. They were then transferred to a growth chamber maintained at 25 °C during the day and 22 °C at night, with 60% humidity and a 16‐h light/8‐h dark photoperiod. After 14 days of cultivation, analyses of growth, physiological parameters, transcriptomics, and metabolomics were conducted on the plants. After 7 days, the plants were transferred to a 1/4‐strength Hoagland liquid medium (Solarbio).^[^ ^54^, ^55^, ^56^ ^]^ In the treatment group, the concentration of GO in the liquid medium was maintained at the same level as that in the SM medium. Water changes were conducted every three days to ensure optimal growth conditions. The plants were cultivated until maturity, after which seeds were harvested.

### Whole Genome Resequencing

High‐quality genomic DNA was extracted from ≈30 mg of seeds from WT, GM, and F1 plants (three biological replicates per group), with F1 plants treated with 0.75 or 1.5 mg‐C/L of GO throughout their lifespan. Each biological sample comprised 30 mg of seeds, with three replicates per treatment group prepared for DNA extraction. DNA was extracted using the DNAsecure Plant Kit (TIANGEN, Beijing, China) according to the manufacturer's instructions. DNA was fragmented into 200–300 bp fragments using a Covaris sonicator, purified using the Agencourt AMPure XP‐Medium Kit (Beckman Coulter, CA, USA), and quantified using the Qubit dsDNA HS Assay Kit (Thermo Fisher). After end repair, A‐tailing, and adaptor ligation with the BGI Plug‐In Adapter Kit (BGI), polymerase chain reaction amplification was conducted on a Bio‐Rad thermal cycler, and product quality was assessed on an Agilent 2100 Bioanalyzer using the Agilent DNA 1000 Reagents (Agilent Technologies, CA, USA). Libraries were sequenced using a MGISEQ‐T7 System (MGI). DNA nanoballs were generated by rolling circle amplification and sequenced using paired‐end 150 bp reads on the DNBSEQ‐T7RS platform (MGI). Detailed data processing steps, including read filtering, alignment, variant detection, and annotation, were conducted using tools such as Soapnuke, BWA, Samtools, GATK, and snpEff, and visualization was performed using the circlize package in R. Full details of the data processing pipeline are provided in the Supporting Information.

### Measurement of Fresh Weight, Root Length, and Root Weight in WT and GM Plants

Fourteen‐day‐old WT and GM plants treated with 0.75 mg‐C/L GO, 1.5 mg‐C/L GO, or without GO were carefully removed from the semi‐solid medium. The roots were rinsed three times with PBS to eliminate the residual medium and gently blotted dry using absorbent paper. Fresh and root weights were measured in groups of five plants, using an electronic balance (Sartorius, Göttingen, Germany). Root length was measured to the nearest millimeter using a ruler.

### RNA‐Seq and Metabolome Analysis

Total RNA was extracted from ≈150 mg of 14‐day‐old WT and GM plants, with or without GO treatment (0.75 mg‐C/L), using TRIzol (Invitrogen, MA, USA), according to the manufacturer's instructions. At least five independent plants were pooled to create a single sample for each treatment and three biological replicates per treatment were used for RNA extraction. RNA integrity was confirmed (RIN > 7.0) before library construction. For each sample, 1.5 µg of RNA was used to construct libraries with the NEBNext® Ultra™ RNA Library Prep Kit for Illumina® (NEB, MA, USA). The mRNA was enriched using oligo (dT) magnetic beads, fragmented, and reverse‐transcribed into cDNA. The cDNA was purified using AMPure XP beads (Beckman Coulter) and assessed for quality using an Agilent 2100 Bioanalyzer (Agilent Technologies). Sequencing was performed on an Illumina NovaSeq 6000 platform (Illumina) with 150 bp paired‐end reads. Raw reads were subjected to quality control using Trimmomatic (v0.33) to remove adapters, low‐quality bases, and reads with >10% ambiguous bases. Clean reads were aligned to the reference genome using STAR (v2.5.2b) and gene expression was quantified as fragments per kilobase of transcript per million mapped reads (FPKM) using HTSeq (v0.5.4 p3). Differential expression analysis was performed using DESeq (v1.10.1) with thresholds of adjusted *p* < 0.05 and |Log_2_FC| > 1, applying the Benjamini–Hochberg method to control the false discovery rate.

WGCNA analysis was performed using the R package WGCNA (v1.72.5) to identify and analyze gene co‐expression networks. FPKM data from 20,389 genes across each treatment group served as the input. A soft‐thresholding power of β = 9 was used to construct an adjacency matrix, resulting in a topological overlap matrix (TOM) with dissimilarities (dissTOM = 1 ‐ TOM) as distance metrics. Modules were identified using a dynamic tree cut with a minimum size of 60, and those with correlations below 0.1 were merged, leading to the final module set.

For the metabolomic analysis, root samples were collected from 14‐day‐old WT and GM plants with or without GO treatment (0.75 mg‐C/L), frozen in liquid nitrogen, and ground. At least ten independent plant roots were pooled to form a single sample for each treatment, and three biological replicates were used for metabolite extraction. ≈50 mg of each powdered sample was mixed with 1200 µL of pre‐cooled (−20 °C) 70% methanol‐water solution containing the internal standard L‐2‐chlorophenylalanine (J&K Scientific, CA, USA). The mixture was vortexed for 30 s every 30 min, repeated six times, and centrifuged at 12 000 rpm for 3 min. The supernatant was filtered through a 0.22 µm membrane and stored in vials for UPLC‐MS/MS analysis. Chromatographic separation was performed using an Agilent SB‐C18 column (1.8 µm, 2.1 mm × 100 mm; Agilent Technologies), and the analysis was conducted on an ExionLC™ AD UPLC system (Sciex, MA, USA) coupled with a QTRAP 4500 tandem mass spectrometer (Sciex). Metabolite analysis was conducted using optimized gradient separation and electrospray ionization parameters (detailed methods are provided in the Supporting Information) to ensure high data quality through periodic quality control and stringent criteria for differential metabolite identification (VIP > 1, |Log_2_FC| > 1). KEGG pathway enrichment analysis was performed based on KEGG pathway analysis. The strengths of the metabolite relationships were inferred from the interaction scores obtained from the STITCH database and visualized using Cytoscape (v3.9.1).

Transcriptome and metabolome data were integrated to evaluate gene‐metabolite correlations. The Pearson correlation coefficient was calculated using R (v4.3.1), selecting gene‐metabolite pairs with |cor| > 0.8 and *p* < 0.05. Gene ontology functional enrichment of differentially expressed genes was conducted using Cytoscape ClueGO (v2.5.10).

### Analysis of JA Contents in Roots

For JA extraction, WT and GM roots were collected at 10, 14, and 20 days, with or without GO treatment (0.75 and 1.5 mg‐C/L). The roots were quickly frozen in liquid nitrogen and ground into fine powder. At least ten independent plant roots were pooled to create a single sample for each treatment, and three biological replicates were used for extraction. A 0.2 g root sample was transferred to a 15 mL centrifuge tube, and 5 mL of an extraction solvent, comprising isopropanol, water, and concentrated hydrochloric acid in a volume ratio of 2:1:0.002, was added. The mixture was agitated at 120 rpm for 30 min at 4 °C. Subsequently, 4 mL of dichloromethane was added to each sample, and the mixture was shaken again at 120 rpm for 30 min at 4 °C. After centrifugation at 13 000 ×g for 5 min at 4 °C, the lower phase was collected and concentrated using a nitrogen evaporator. The final extract was dissolved in 0.2 mL of methanol and filtered through a 0.22 µm membrane filter before analysis. JA was quantified using an HPLC‐MS system comprising a liquid chromatography unit (Shimadzu, Kyoto, Japan),^[^ ^9^ ^]^ a mass spectrometer (API 4500 QTrap, Applied Biosystems, CA, USA), and a C18 column (Kinetex EVO, 2.6 µm, 2.1 × 100 mm^2^, Phenomenex, CA, USA). The JA standards were obtained from Macklin. The detailed analytical conditions are provided in Supporting Information.

### Ultrastructural Examination of Chloroplasts by TEM

TEM (80 kV, Hitachi, Tokyo, Japan) was conducted to examine the ultrastructure of 14‐day‐old *A. thaliana* leaves from WT and GM plants with or without GO treatment (0.75 mg‐C/L). Fresh leaf tissue (≈1 mm^3^) was fixed in 2.5% glutaraldehyde at 4 °C for 12 h, then post‐fixed with 1% osmium tetroxide (SPI Supplies, DE, USA) for 1 h, followed by three 15‐min washes in 0.1 M PBS (pH 7.4; Sigma–Aldrich, MO, USA). Dehydration was conducted using an ethanol gradient (30%, 50%, 70%, 90%, and 100%), followed by two 20‐min treatments with 100% acetone (Sigma–Aldrich). The samples were infiltrated with Spurr's resin (1:1 for 3 h, 1:3 for 4 h; SPI Supplies) at 37 °C, embedded in pure Spurr's resin at 37 °C overnight, and then polymerized at 70 °C for 12 h. Ultrathin sections (70–90 nm) were cut on a Leica UC7 ultramicrotome (Leica Microsystems, Wetzlar, Germany), mounted on copper grids (Ted Pella Inc., CA, USA), and stained with uranyl acetate and lead citrate (Ted Pella Inc.).

### Measurement of Photosynthetic Parameters

Chlorophyll fluorescence measurements were conducted using a modulated fluorometer (Soliense, NY, USA) following a 30‐min dark adaptation to ensure fully open PSII reaction centers on 14‐day‐old WT and GM plants, with or without GO treatment (0.75 and 1.5 mg‐C/L). Measurement parameters were set: PS threshold at 50, Fm factor and F factor at 1.000, Yield filter at 3, and PAR at 80 µmol photons m⁻^2^ s⁻¹. A saturating flash intensity of 2500 µmol photons m⁻^2^ s⁻¹ was employed. Data acquisition and analysis of Fv/Fm and Y(II) were performed using the IMAGING WIN software (v41a). In treatments requiring ALA, 1 µm ALA (MedChemexpress, NJ, USA) was added to GO‐supplemented and control media. The ALA stock solution was prepared by combining 10 µL of a 25 mg mL^−1^ DMSO stock with 490 µL of PEG300, followed by the addition of 50 µL of Tween‐80 to enhance solubility, and then dilution with 450 µL of 0.9% (w/v) saline to a final volume of 1 mL, into which 5 mg of ALA was introduced.

### Whole Genome Bisulfite Sequencing

High‐quality genomic DNA was extracted from ≈30 mg of WT, GM, and F1 seeds with and without GO treatment (0.75 and 1.5 mg‐C/L) using the DNAsecure Plant Kit (TIANGEN). Each biological sample comprised 30 mg of seeds with three replicates prepared for DNA extraction per treatment group. The extracted DNA was fragmented to an average size of ≈250 bp using a Bioruptor (Diagenode, Liege, Belgium). This fragmented DNA underwent end repair, 3′ A‐tailing, and adapter ligation, followed by bisulfite conversion with the EZ DNA Methylation‐Gold Kit (ZYMO, CA, USA). Target fragments were size‐selected using 2% agarose gel electrophoresis, recovered using a QIAquick Gel Extraction Kit (Qiagen, Hilden, Germany), and amplified using polymerase chain reaction to create libraries. Library construction was performed using an MGIEasy whole‐genome bisulfite library preparation kit (MGI). After quality checking, the library was amplified using phi29 to generate DNA nanoballs, which were loaded onto a high‐density DNA nanoball chip and sequenced using circumferential probe‐anchored polymerase amplification on a DNBSEQ‐G400 sequencer (BGI) with PE150 sequencing. Initial sequence reads were processed using SOAPnuke (v1.5.6) for quality control, removing reads with > 10% N content or > 10% bases below Q20. Clean reads were aligned to the reference genome using BSMAP (v2.74) to compute alignment rates and bisulfite conversion efficiency.

Methylation levels were determined as the proportion of reads showing methylation at each cytosine, calculated as follows: Methylation level = Nm / (Nm + Nn), where Nm and Nn represent the number of reads supporting methylated and non‐methylated cytosines, respectively. Whole‐genome methylation levels were calculated as mean values within 100 kb windows for each chromosome and visualized using the circlize package (v0.4.16) in R (v4.3.1).

Differentially methylated regions (DMRs) were identified by analyzing windows containing at least five CG (or CHG/CHH) sites across samples. Regions with significant differences in methylation (|Log_2_FC| > 1, Fisher's exact test, *p*<0.05) were designated as DMRs. Adjacent DMRs exhibiting similar methylation changes were merged into contiguous regions; otherwise, they were treated as separate regions. The degree of differential methylation was calculated using the following formula: degree of difference = Log_2_ (Rm1/Rm2), where Rm1 and Rm2 represent methylation levels in samples 1 and 2, respectively. If either Rm1 or Rm2 was equal to zero, they were replaced with 0.001 in the calculations. The DMR analysis between groups was further refined and evaluated using metilene (v0.2‐7), which facilitated the identification of significant differential methylation across genomic regions.

### Statistical Analysis

Each treatment group comprised at least 30 plants, with 5 or 10 independent plants pooled per biological sample for transcriptome or root metabolome analyses. For DNA extraction, 30 mg of seeds per treatment was combined into a single biological sample. Three biological replicates were prepared for the extraction and analysis of each treatment. *p* values were calculated using independent sample two‐sided *t*‐tests in SPSS (v25).

## Conflict of Interest

The authors declare no conflict of interest.

## Supporting information



Supporting Information

## Data Availability

The data that support the findings of this study are openly available in NCBI at https://www.ncbi.nlm.nih.gov/sra/PRJNA1192418, reference number 1192418.
