# Supplementary material for: Accounting for ALA Natural Mutations Enhances the Efficiency of Graphene Oxide Nanopriming in Bar‐Modified Arabidopsis
Source: Adv Sci (Weinh). 2025 Jun 5;12(32):e00058. doi: 10.1002/advs.202500058 (PMC12407378; doi:10.1002/advs.202500058)
Supplement: Supplementary file 1 — Supporting Information [file ADVS-12-e00058-s001.docx]

Supporting Information

Accounting for ALA Natural Mutations Enhances the Efficiency of Graphene Oxide Nanopriming in *Bar*-Modified *Arabidopsis*

*Yining Wu^a^, Rui Sun^a^, Yueting Cui^a^, Jun Qiao^b^, Chengdong Zhang^a^**

* Corresponding author: Chengdong Zhang

**Email:**  zhangchengdong@bnu.edu.cn;

**This PDF file includes:**

Supporting text

Figures S1 to S8

Tables S1 to S2

SI References

**Supporting Information Text**

*Characterization of* *graphene oxide (GO)*: The morphology of monolayer GO powder was characterized using a Transmission Electron Microscope (TEM, JEM-F200, JEOL) operated at 200 kV. The structural features and defect characteristics of GO were analyzed using Raman spectroscopy (LabRAM HR Evolution, Horiba) with a 532 nm excitation wavelength. The crystal structure of GO was determined by X-ray Diffraction (XRD, Ultima IV, Rigaku) with a tube voltage of 40 kV, a current of 40 mA, and a copper target (wavelength 0.154 nm). X-ray Photoelectron Spectroscopy (XPS, K-Alpha, Thermo Scientific) was employed to study the electronic states and chemical composition of GO. XPS measurements were conducted with a spot size of 400 μm, a working voltage of 12 kV, and a filament current of 6 mA. Full-spectrum scans were performed with a pass energy of 150 eV and a step size of 1 eV, while narrow scans used a pass energy of 50 eV and a step size of 0.1 eV.

*Total Organic Carbon (TOC) testing procedure:* The TOC content was measured using a TOC-L CPH analyzer (Shimadzu) under the following conditions: the furnace temperature was set to 680 °C, with high-purity synthetic air as the carrier gas at a flow rate of 150 mL/min. The sample vial was an ASI-L 24 mL, and a standard diameter injection-spraying needle was employed.

*Construction of transgenic vectors for bar and GFP genes and genetic transformation in Arabidopsis thaliana (**A. thaliana)*: To construct the *bar* gene overexpression vector, specific primers were designed and synthesized (forward primer: 5'-cagtggtctcacaacatgagcccagaacgacgccc-3'; reverse primer: 5'-cagtggtctcatacatcaaatctcggtgacgggca-3'). Polymerase chain reaction (PCR) amplification of the target fragment was conducted in a 50 μL reaction mixture containing 34 μL of ddH₂O, 5 μL of 10× PCR buffer, 4 μL of 25 mM MgCl₂, 2 μL of 10 mM dNTP mix, 2 μL of 10 μM forward primer, 2 μL of 10 μM reverse primer, 1 U of Taq DNA polymerase, and 1 μL of template DNA. The PCR conditions were as follows: initial denaturation at 94 °C for 5 min, followed by 30 cycles at 94 °C for 30 s, 50 °C for 45 s, and 72 °C for 33 s, with a final extension at 72 °C for 10 min and a hold at 16 °C. The 552 bp product was excised from a 1.5% agarose gel and purified. The purified fragment was then digested with BsaI/Eco31I and ligated into the pBWA(V)KS-ccDB vector, which had been similarly digested. The ligation reaction was transformed into *Escherichia coli*, plated on kanamycin-selective agar, and positive clones were confirmed by colony PCR.

For the *GFP* gene, primers were designed with the following sequences: forward: 5'-cagtggtctcacaacatgtgtatcgtgaagggcga-3' and reverse: 5'-cagtggtctcatacatcagtagagctcgtccatgc-3'. The PCR amplification was performed in a 50 μL reaction mix consisting of 34 μL of ddH₂O, 5 μL of 10× PCR buffer, 4 μL of 25 mM MgCl₂, 2 μL of 10 mM dNTP mix, 2 μL of 10 μM forward primer, 2 μL of 10 μM reverse primer, 1 U of Taq DNA polymerase, and 1 μL of template DNA. The PCR program included an initial denaturation at 94 °C for 5 min, followed by 30 cycles of 94 °C for 30 s, 50 °C for 45 s, and 72 °C for 43 s, concluding with a final extension at 72 °C for 10 min. The resulting 720 bp band was purified, digested with BsaI/Eco31I, and ligated into the pBWA(V)KS-ccDB vector. The ligation reaction was transformed into *Escherichia coli*, plated on a kanamycin-selective medium, and verified by colony PCR.

To generate a vector for the dual expression of *bar* and *GFP* genes, individual overexpression vectors were prepared by digesting them with BsmBI/Esp3I and performing ligation using T4 ligase. The 20 μL ligation reaction included 8 μL of ddH₂O, 2 μL of 10× buffer, 1 μL of BsmBI/Esp3I enzyme, 1 μL of T4 ligase, 4 μL of the *bar* overexpression construct, and 4 μL of the *GFP* overexpression construct. The ligation protocol was as follows: an initial incubation at 37 °C for 20 min, followed by 5 cycles of 37 °C for 10 min and 20 °C for 10 min, concluding with a final incubation at 37 °C for 20 min and inactivation at 80 °C for 5 min. After transformation into *Escherichia coli*, plasmids were extracted and verified for further applications.

For the transformation into *A. thaliana*, 1 μL of the dual expression plasmid was introduced into *Agrobacterium tumefaciens* strain GV3101 via electroporation. The transformed cells were plated on kanamycin-selective medium and incubated at 30 °C in the dark for 48 h. To confirm successful transformation, colony PCR was performed using specific primers (forward primer: 5'-atgagcccagaacgacgcc-3' and reverse primer: 5'-tcaaatctcggtgacgggca-3'). Positive colonies were selected for subsequent floral dip transformation experiments.

Wild-type (WT) seeds of *A. thaliana* were sown and covered to maintain humidity. After developing two true leaves, the seedlings were transplanted to pots, covered for 3 days, and uncovered. One month later, primary inflorescences were trimmed to encourage additional flowering. *Agrobacterium tumefaciens* GV3101 containing the dual vector was cultured to an OD600 of 0.8 with 0.02% Silwet-77, and the inflorescences were dipped in this suspension for 2 s, covered to maintain over 90% humidity, and incubated at 25 °C in darkness for 24 h. This dip was repeated weekly for 2 cycles. Plants were grown at 23 °C under 16 h of light and 8 h of darkness until the seeds matured. Harvested siliques were dried at 37 °C for 24 h, and the seeds were sieved and sterilized with 75% ethanol, then rinsed with sterile water. The seeds were placed on a kanamycin-selective medium, stratified at 4 °C for 2 days, then grown at 23 °C under 16 h of light and 8 h of darkness for 14 days. Kanamycin-resistant seedlings were transplanted to soil under the same conditions. After 20 days, genomic DNA was extracted using the DNAsure Plant Kit (TIANGEN) and analyzed by PCR for transgene presence. Confirmed transgenic plants were grown to maturity, and T1 seeds were collected from dried siliques. The 35S::*bar* seeds used in this study were derived from T1 generation plants.

*GFP fluorescence observation*: *GFP* fluorescence in 14-day-old WT and genetically modified (GM) *A. thaliana* was observed using confocal laser scanning microscopy on an Olympus FV1000 microscope (Olympus) with an excitation wavelength of 488 nm and an emission wavelength of 509 nm.

*Whole genome resequencing data processing*: Whole genome resequencing data were processed to generate paired-end FASTQ files. Soapnuke (Version: V2.2.6) was utilized to filter out adapter contamination and low-quality reads, resulting in high-quality clean data for alignment analysis. The alignment process employed BWA software (Version: V0.7.1) with the Mem algorithm to map the clean data to the reference genome, generating a SAM format alignment file. Samtools (Version: V1.9) was then used for sorting and deduplication. Variant detection was performed using GATK (Version: V4.2.6.1), yielding extensive variant data. Stringent filtering criteria were applied, including QD < 2.0, MQ < 40.0, FS > 60.0, QUAL < 30.0, MQRankSum < -12.5, and ReadPosRankSum < -8.0, along with settings of -clusterSize 2 and -clusterWindowSize 5. High-quality single-nucleotide polymorphisms (SNPs) and insertions/deletions (Indels) data were obtained for downstream analysis following these filtering steps. In the variant annotation phase, SNPs and Indels were annotated using snpeff (Version: V4.3.1) based on the physical locations of the detected variants. SNPs and Indels distributions across chromosomes were calculated as mean values within 100 kb windows and visualized using the circlize package (Version: 0.4.16) in R (Version: 4.3.1).

*Measurement of fresh weight, root length, and root weight in WT and GM plants:* 14-day-old WT and GM plants treated with 0.75 mg-C/L GO, 1.5 mg-C/L GO, or without GO were carefully removed from the semi-solid medium. Roots were rinsed three times with PBS to eliminate residual medium and gently blotted dry using absorbent paper. Fresh weight and root weight were measured for groups of five plants using an electronic balance (Sartorius). Root length was measured with a ruler to the nearest millimeter.

*Experimental conditions and data analysis for metabolomics:* The analytical method was modified as follows:^[1]^ the mobile phase consisted of solvent A (ultrapure water with 0.1% formic acid) and solvent B (acetonitrile with 0.1% formic acid). The gradient program began with 95% A and 5% B, linearly increasing to 5% A and 95% B over 9 min, held for 1 min, then returning to initial conditions in 1.1 min, followed by 2.9 min of equilibration. The flow rate was set to 0.35 mL/min, the column temperature to 40 °C, and the injection volume to 2 μL. Electrospray ionization source parameters included a source temperature of 550 °C, ion spray voltage of 5500 V (positive mode) or -4500 V (negative mode), with gas flows set at 50 psi for ion source gas I, 60 psi for ion source gas II, and 25 psi for curtain gas. Collision-activated dissociation was set to high, and multiple reaction monitoring mode transitions were optimized for each metabolite to capture specific ion signals. Data processing was performed using Analyst software (Sciex, Version: 1.6.3), where peak areas were integrated and normalized across samples. Quality control samples were analyzed every ten samples to monitor repeatability, maintaining a coefficient of variation below 0.5 to ensure data stability. Differential metabolites were identified based on a variable importance in projection (VIP) score > 1, derived from orthogonal partial least squares discriminant analysis (OPLS-DA), and | Log_2_ Fold Change [FC]| > 1. The VIP score reflects the contribution of each metabolite to group discrimination within the model, with VIP > 1 indicating significant differential metabolites. These metabolites were subsequently analyzed through Kyoto Encyclopedia of Genes and Genomes (KEGG) pathway enrichment. Metabolite interactions were studied using the STITCH database (http://stitch.embl.de/) and then re-visualized in Cytoscape (Version: 3.9.1).

*Measurement of antioxidant enzyme activities and stress-related metabolites*: To evaluate antioxidant enzyme activities and the accumulation of stress-related metabolites, 0.1 g of 14-day-old *A. thaliana* seedlings was collected. All biochemical parameters were quantified using commercial assay kits strictly following the manufacturers’ protocols. Catalase (CAT) activity was measured using a Hydrogen Peroxide Assay Kit (Shanghai Beyotime Biotechnology Co., Ltd.). Superoxide dismutase (SOD) activity was determined using the Total SOD Activity Assay Kit (NBT method; Shanghai Beyotime Biotechnology Co., Ltd.). Glutathione reductase (GR) activity was analyzed using a GR Activity Assay Kit (Beijing Solarbio Science & Technology Co., Ltd.). Total antioxidant capacity was evaluated using a Total Antioxidant Capacity Assay Kit (Beijing Solarbio Science & Technology Co., Ltd.). malondialdehyde (MDA) content was determined with an MDA Assay Kit (Beijing Solarbio Science & Technology Co., Ltd.). Proline content was assessed using a Proline Content Assay Kit (Beijing Solarbio Science & Technology Co., Ltd.), and soluble sugar levels were quantified using a Plant Soluble Sugar Assay Kit from the same supplier. For normalization, protein concentrations were determined using an Enhanced BCA Protein Assay Kit (Shanghai Beyotime Biotechnology Co., Ltd.).

*Analysis of jasmonic acid (JA), indole-3-acetic acid (IAA), and abscisic acid (ABA) by liquid chromatography-mass spectrometry (HPLC-MS):* The extraction method for IAA and ABA was the same as that used for JA, as described in the main text. Quantification of JA, ABA, and IAA was performed using an HPLC system (Shimadzu) coupled with a mass spectrometer (API500 QTrap, Applied Biosystems) and a C18 column (Kinetex 2.6 μm EVO, 2.1 × 100 mm², Phenomenex). Standards for JA, IAA, and ABA were obtained from Macklin. The analytical method was as follows:^[2]^ the mobile phases included 0.1% formic acid in water (Phase A) and 0.1% formic acid in methanol (Phase B), with a flow rate of 0.3 mL/min. The gradient started at 25% B, increased to 60% by 9.0 min, held at 60% until 10.0 min, then reduced to 10% at 10.1 min and held until 15.0 min. The column temperature was maintained at 40 °C. Autosampler settings comprised a 200 µL rinse volume, a 52 mm needle stroke, a 35 µL/s rinsing speed, a 5.0 µL/s sampling speed, and a 25-min purge. MRM mode was used with optimized declustering potential (DP) and collision energy (CE) for each compound: SA (Q1/Q3: 136.800/92.900, DP: -25 V, CE: -34.600 V) and JA (Q1/Q3: 208.500/165.100, DP: -101 V, CE: -17.700 V).

*Metabolite network analysis*: Roots from 14-day-old plants of the 35S::*bar* and WT groups treated with 0.75 mg-C/L GO were analyzed using metabolomics. Following this analysis, terpenoids and lignans/coumarins metabolite-metabolite network analysis was conducted utilizing the STITCH database, with relationship strength inferred from STITCH interaction scores and visualized in Cytoscape (Version: 3.9.1).

*Z-Score calculation for gene ontology term enrichment analysis*: The Z-score for each gene ontology term was calculated based on the mean Log_2_FC of genes associated with that term using the formula:

$$\frac{Mean\left( {Log}_{2}{FC}_{term} \right)-Mean\left( {Log}_{2}{FC}_{all genes} \right)}{SD\left( {Log}_{2}{FC}_{all genes} \right)}$$

Where: Mean (Log_2_FC_term_) represents the mean Log_2_FC of genes associated with the specific gene ontology term; Mean (Log_2_FC_all genes_) represents the mean Log_2_FC of all genes; SD (Log_2_FC_all genes_) is the standard deviation of Log_2_FC across all genes.


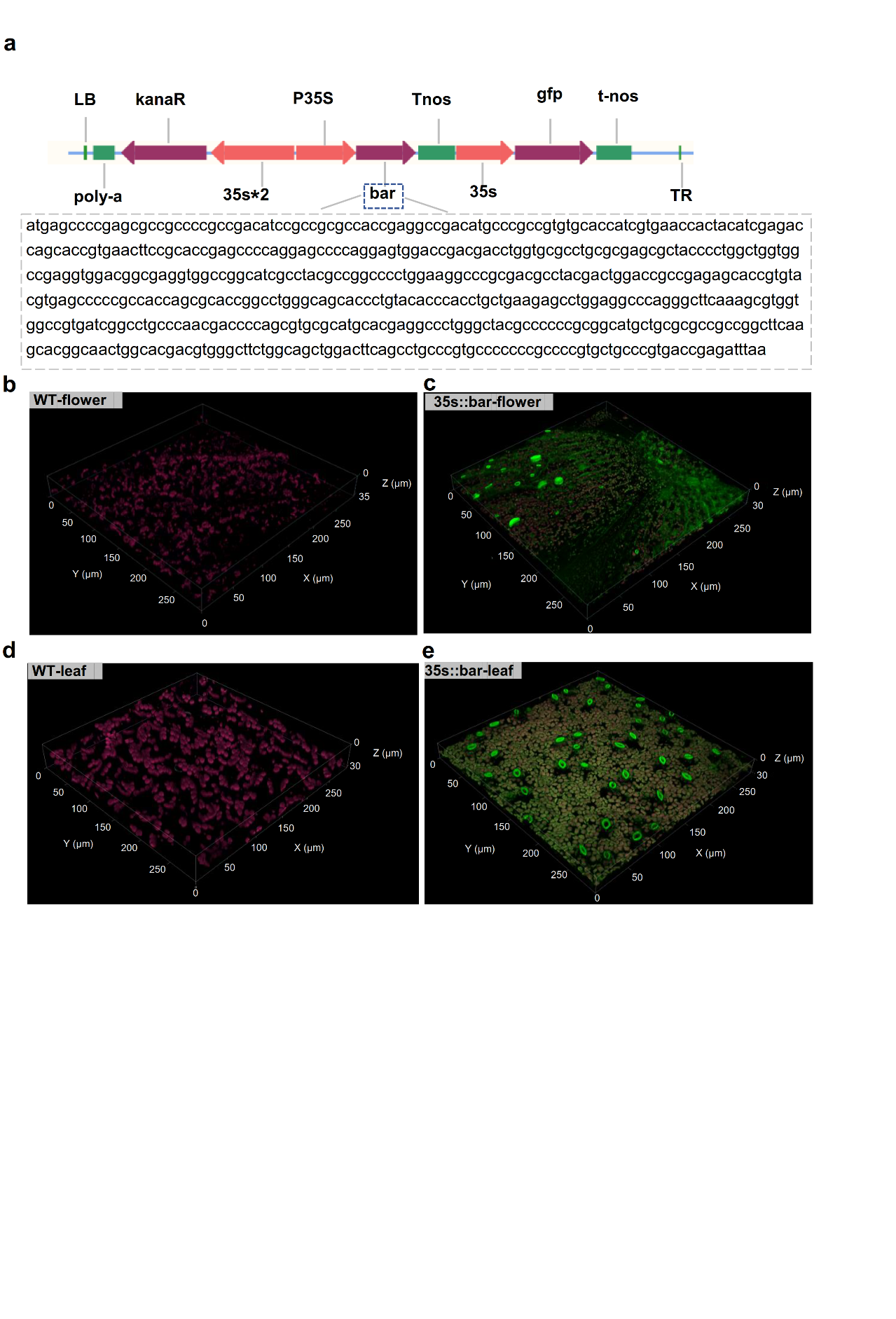


Figure S1. Genetic construct and fluorescence imaging of *bar* gene expression in WT and GM *A. thaliana*. (a) Schematic of the genetic construct: The construct includes the *bar* gene (with its specific sequence) regulated by the 35S promoter, the *kana* gene for kanamycin resistance, and the *GFP* gene functioning as a reporter. (b-e) 3D fluorescence images showing *bar* expression in WT and 35S::*bar* plants. (b) WT flower, (c) 35S::*bar* flower, (d) WT leaf, and (e) 35S::*bar* leaf.


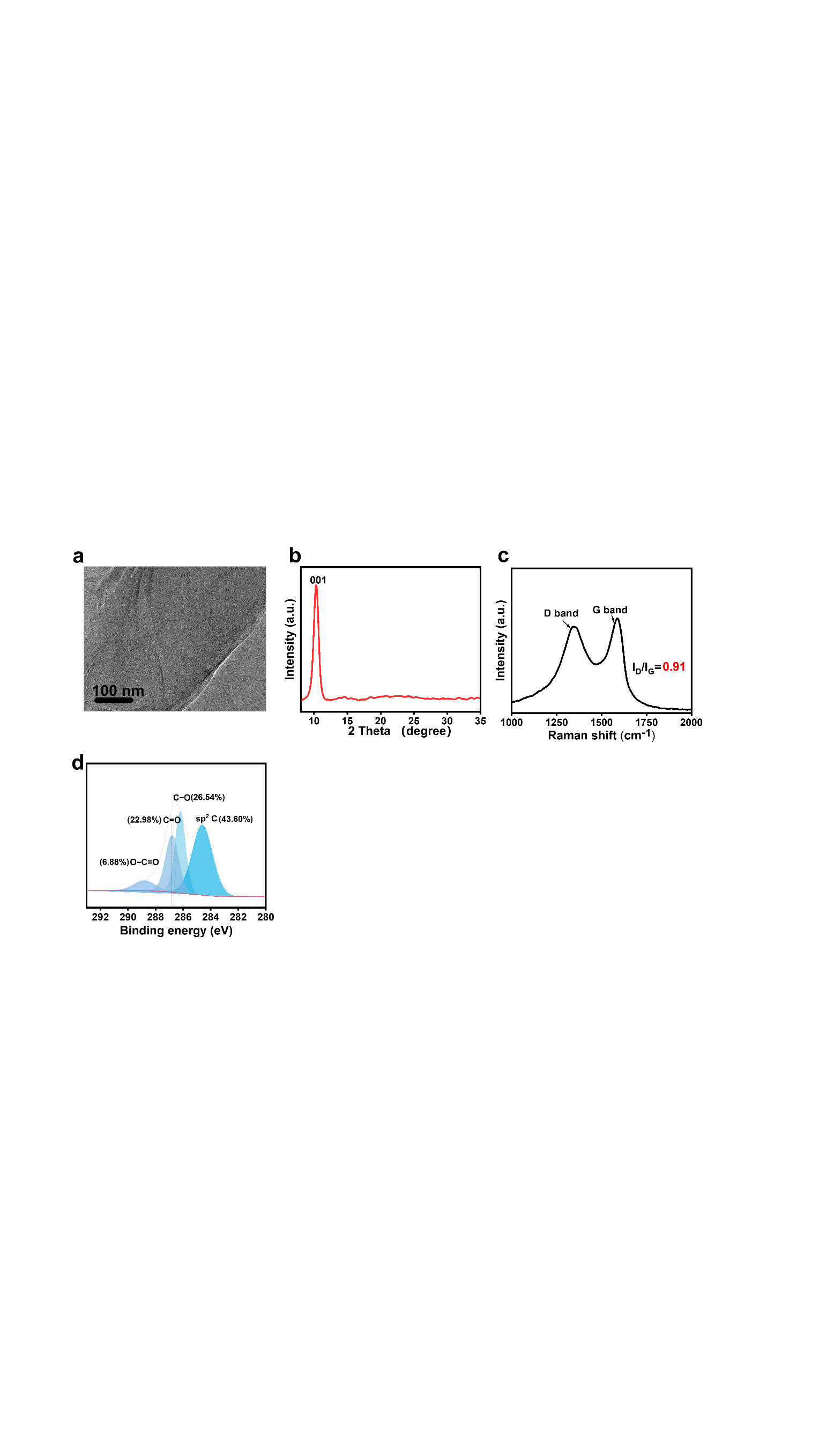


Figure S2. Characterization of GO's structure and surface. (a) TEM image of GO sheets. (b) XRD pattern of GO. (c) Raman spectrum of GO. (d) XPS spectrum of GO.


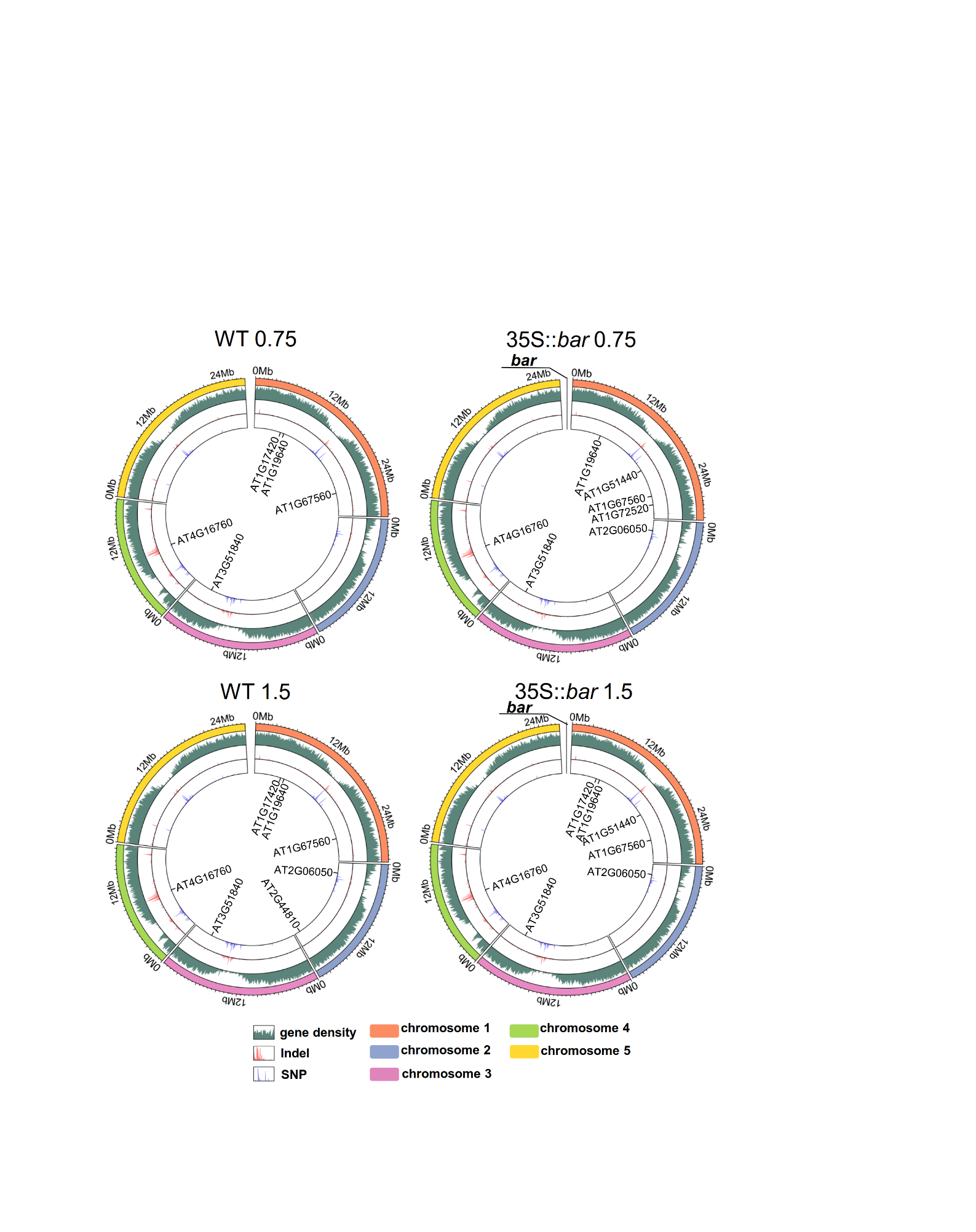


Figure S3. Distribution of genes, SNPs, and Indels across each chromosome in WT and GM A. thaliana under 0.75 and 1.5 mg-C/L GO treatments. A Circos plot illustrates the distribution of SNPs and Indels on each chromosome. The colored sections of the outer ring represent different chromosomes. The green inner ring indicates gene density throughout the genome. Red and blue lines depict the densities of Indels and SNPs, respectively. Density is defined as the number of SNPs or Indels per 100 kb. The center labels identify the genes associated with α-linolenic acid (ALA) metabolism within the genome.


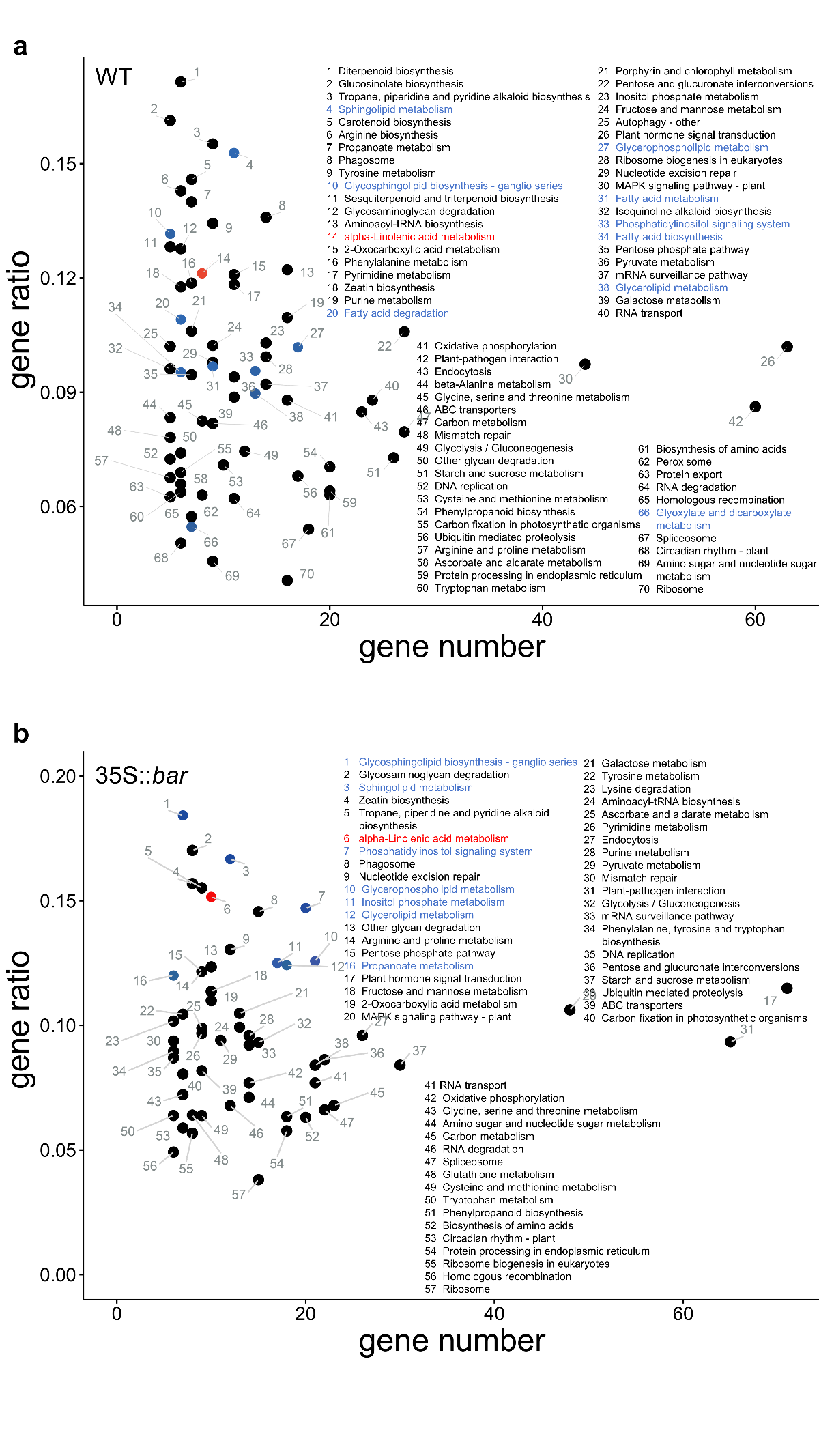


Figure S4. Scatter plots of KEGG enriched pathways (gene number >5) based on genomic mutations identified in (a) WT and (b) GM ancestors through resequencing. The y-axis represents the ratio of gene numbers to the total number of genes in each pathway, while the x-axis indicates the number of genes. The numbers correspond to specific pathways listed alongside the plot. Pathways highlighted in red represent ALA metabolism, while those in blue are related to the ALA metabolic pathway, indicating their roles in lipid metabolism and their connection to ALA metabolism.


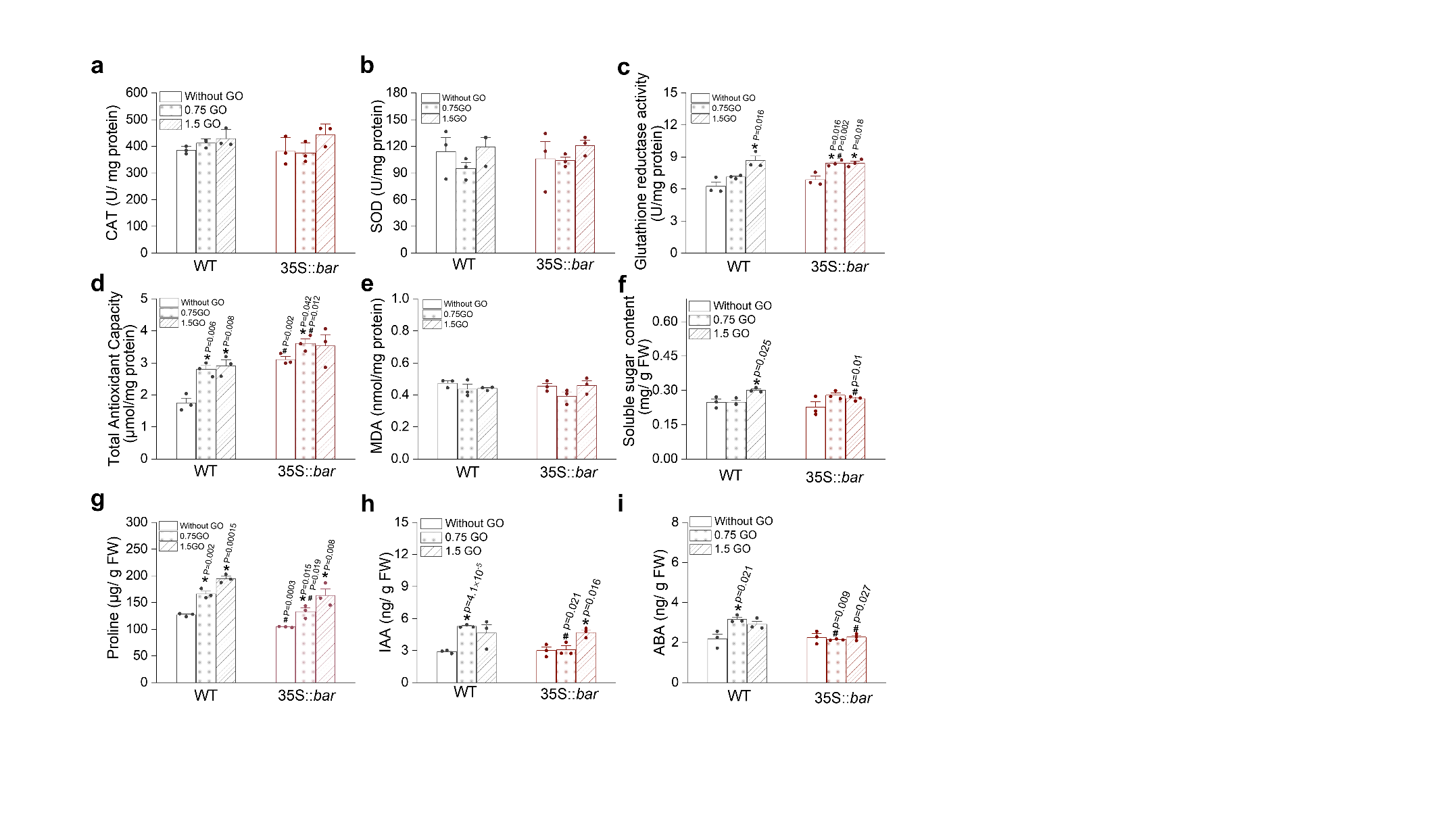


Figure S5. Growth enhancement in 14-day-old WT and GM (35S::bar) plants under GO priming. Measurements of (a) CAT activity; (b) SOD activity; (c) glutathione reductase activity; (d) total antioxidant capacity; and (e) MDA content. The contents of (f) soluble sugar, (g) proline, (h) IAA, and (i) ABA. * indicates significant differences within the same genotype compared to the condition without GO, while # indicates significant differences between GM and WT under the same treatment. P values are included in the figure, with a sample size of n = 3 for each treatment, consisting of three biological replicates. Independent two-sided t-tests were performed.


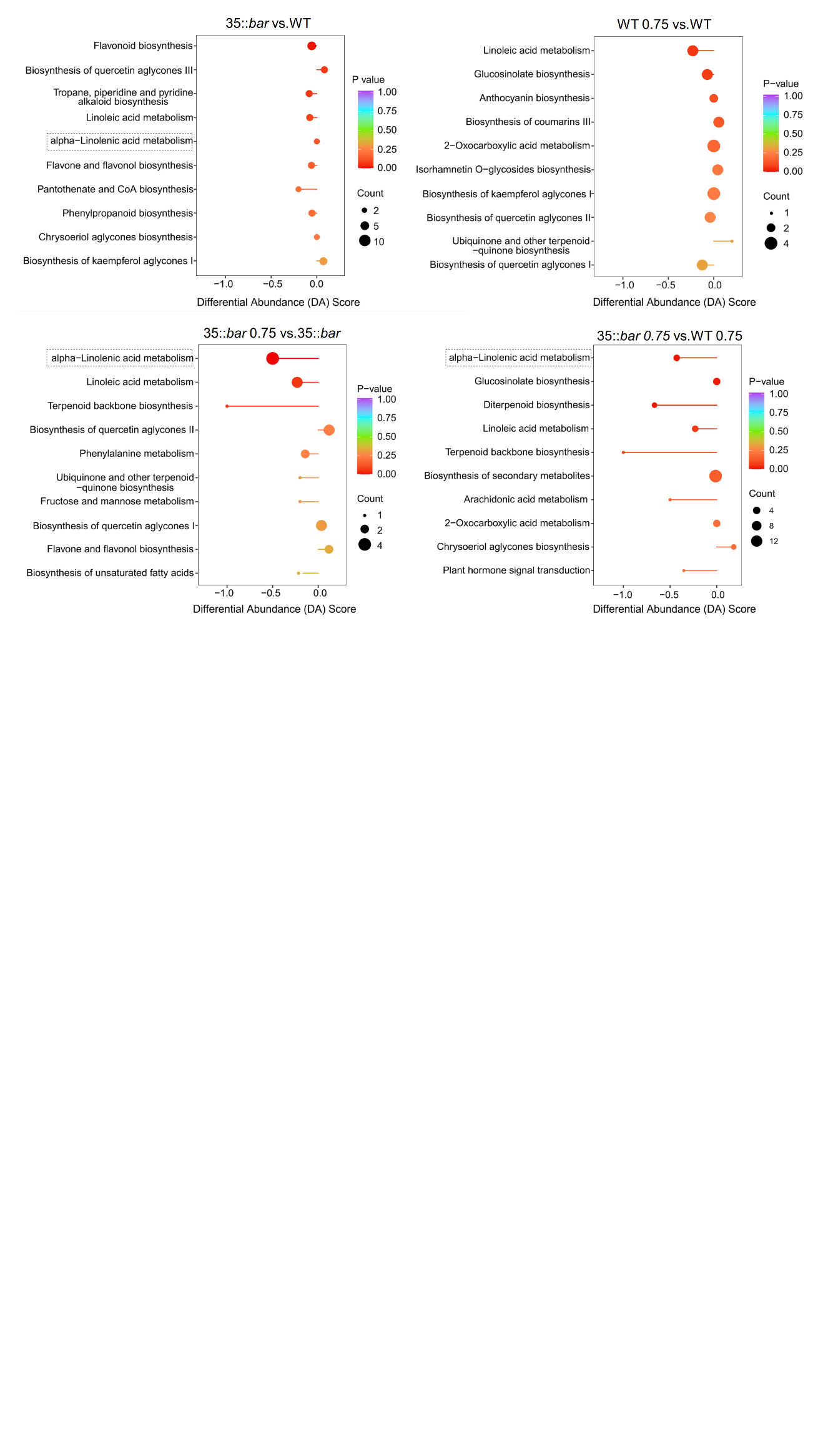
Figure S6. Bubble plots illustrate the differential abundance (DA) scores of the top 10 enriched metabolic pathways derived from the KEGG analysis of differential metabolites identified in the metabolomics analysis of 14-day-old plant roots. The comparisons include (a) 35S::bar vs. WT, (b) WT 0.75 vs. WT, (c) 35S::bar 0.75 vs. 35S::bar, and (d) WT 0.75 vs. 35S::bar 0.75. The y-axis displays the enriched pathways, while the x-axis represents the DA scores. The sizes of the bubbles indicate pathway counts, and the colors of the bubbles signify P values, with more significant pathways indicated by warmer colors. A longer line signifies a greater deviation of the DA score from zero.


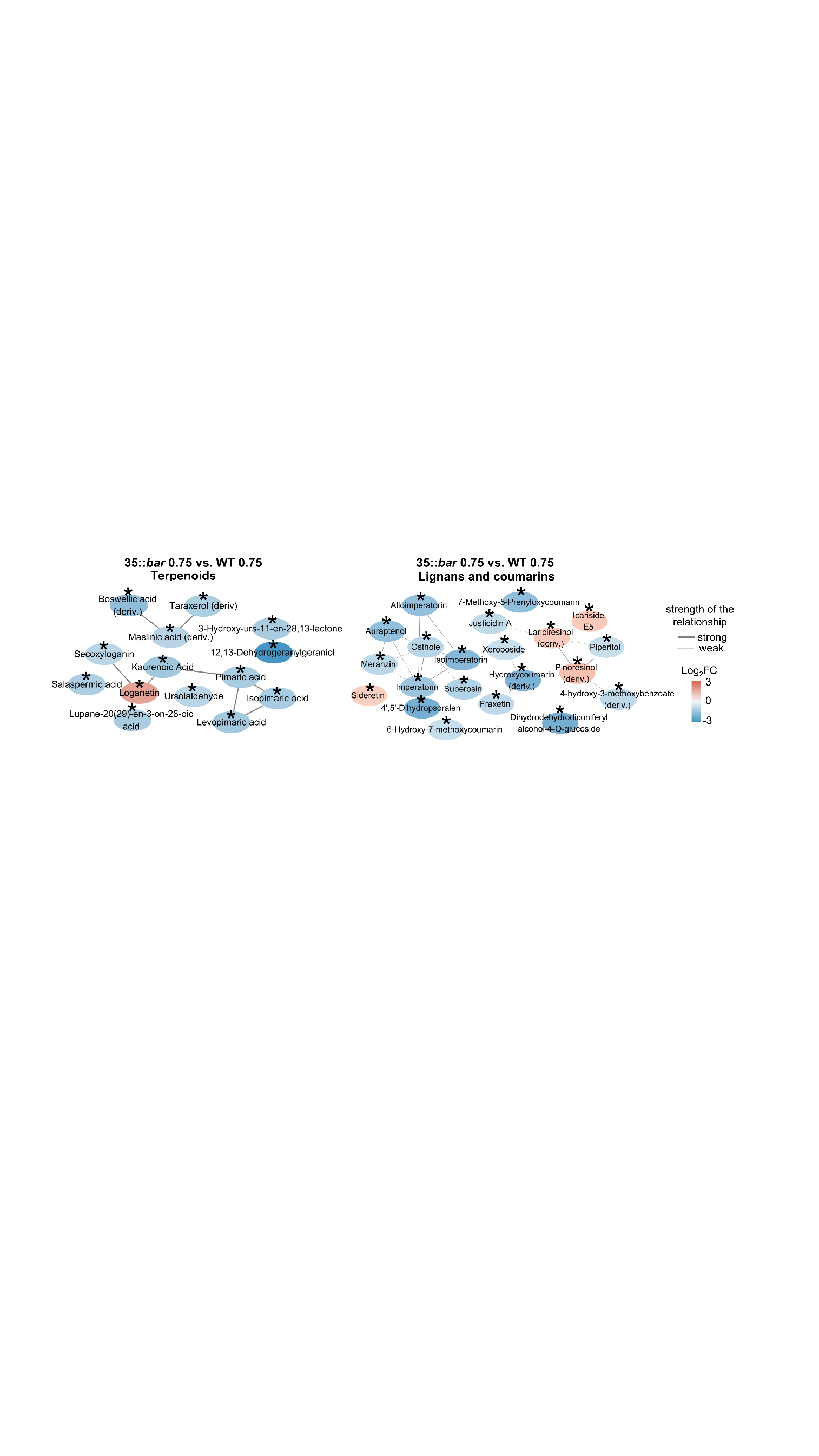


Figure S7. Metabolite-metabolite network analysis of differentially accumulated (a) terpenoids and (b) lignans/coumarins identified from the metabolomics analysis of 14-day-old plant roots under 0.75 mg-C/L GO treatment in 35S::bar versus WT comparisons. Metabolites marked with * indicate |Log2FC| > 1 and variable importance in projection (VIP) > 1. The strength of metabolite relationships was inferred from STITCH interaction scores.


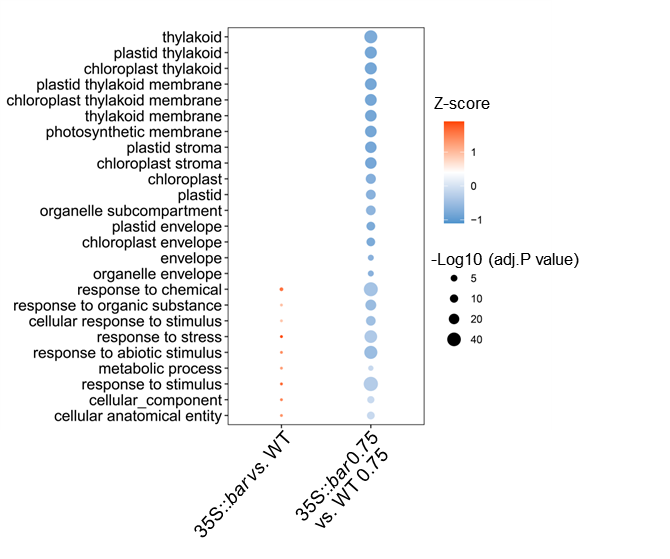


Figure S8. The top 25 pathways significantly downregulated through transcriptomic analysis of GM compared to WT A. thaliana under a 0.75 mg-C/L GO treatment. A Z-score greater than 0 indicates an overall upregulation of the pathway, while a Z-score less than 0 represents an overall downregulation.

Table S1. Abbreviations of lipid metabolites shown in the lipid metabolite network analysis (Figure 2h) under 0.75 mg-C/L GO treatment.

| Abbreviation | Full Name |
| --- | --- |
| 12-OPDA | 12-Oxo-phytodienoic acid |
| 13-HODE | 13(S)-Hydroxyoctadeca-9Z,11E-dienoic acid (13(S)-HODE) |
| 5-oxo-ETE | 5-Oxo-6E,8Z,11Z,14Z-eicosatetraenoic acid |
| 9R,10S-EpOME | (9R,10S)-(12Z)-9,10-Epoxyoctadecenoic acid (9R,10S-EpOME) |
| 9-HODE | 9S-Hydroxy-10E,12Z-octadecadienoic acid |
| LysoPC 15:1 | Lysophosphatidylcholine 15:1 |
| LysoPC 20:2 | Lysophosphatidylcholine 20:2 |

Table S2. *P*-values comparing JA levels in the roots of WT and GM plants exposed to 0.75 or 1.5 mg-C/L GO (Figure 2i)

| Day Group | 10 day | | 14 day | 20 day |
| --- | --- | --- | --- | --- |
| WT0.75 vs. WT | | *P*=0.016 | *P*=0.078 | *P*=0.01 |
| WT1.5 vs. WT | | *P*=0.004 | *P*=0.068 | *P*=0.122 |
| GM0.75 vs. GM | | *P*=0.185 | *P*=0.006 | *P*=0.001 |
| GM1.5 vs. GM | | *P*=0.037 | *P*=0.002 | *P*=0.015 |
| GM vs. WT | | *P*=0.263 | *P*=0.883 | *P*=0.505 |
| GM0.75 vs. WT0.75 | | *P*=0.011 | *P*=0.001 | *P*=0.012 |
| GM1.5 vs. WT1.5 | | *P*=0.000133 | *P*=0.000466 | *P*=0.053 |

**Reference**

[1] H. Jiang, Y. Shi, J. Liu, Z. Li, D. Fu, S. Wu, M. Li, Z. Yang, Y. Shi, J. Lai, X. Yang, Z. Gong, J. Hua, S. Yang, *Nat. Plants* **2022**, *8*, 1176.

[2] Y. Cui, K. Wang, C. Zhang, *ACS Nano* **2024**, *18*, 10829.
